# A review on occurrence of emerging pollutants in waters of the MENA region

**DOI:** 10.1007/s11356-021-16558-8

**Published:** 2021-10-19

**Authors:** Imen Haddaoui, Javier Mateo-Sagasta

**Affiliations:** 1 Regional Center of Agricultural Research, Gafsa street, 9100, Sidi Bouzid, Tunisia; 2 Non-Conventional Water Valuation Research Laboratory (LR VENC), INRGREF, Hedi EL Karray Street, El Menzah IV, 1004 Tunis, Tunisia; 3 International Water Management Institute, Battaramulla, Sri Lanka

**Keywords:** Fresh water, Raw wastewater, Treated wastewater, Irrigation, Emerging pollutants, MENA region

## Abstract

Little is known about the occurrence of emerging pollutants (EPs) in waters in the Middle East and North Africa (MENA) region despite the extensive use of low-quality water there. Available data dealing with the sources, occurrence and removal of EPs within the MENA region in different categories of water is collected, presented and analyzed in this literature review. According to the collected database, the occurrence and removal efficiency of EPs in the water matrix in the MENA region is available, respectively, for 13 and six countries of the 18 in total; no available data is registered for the rest. Altogether, 290 EPs have been observed in different water matrices across the MENA countries, stemming mainly from industrial effluents, agricultural practices, and discharge or reuse of treated wastewater (TWW). Pharmaceutical compounds figure among the most frequently reported compounds in wastewater, TWW, surface water, and drinking water. Nevertheless, pesticides are the most frequently detected pollutants in groundwater. Worryingly, 57 cases of EPs have been reported in different fresh and drinking waters, exceeding World Health Organization (WHO) and European Commission (EC) thresholds. Overall, pharmaceuticals, organic compounds, and pesticides are the most concerning EP groups. The review revealed the ineffectiveness of treatment processes used in the region to remove EPs. Negative removals of some EPs such as carbamazepine, erythromycin, and sulfamethoxazole were recorded, suggesting their possible accumulation or release during treatment. This underlines the need to set in place and strengthen control measures, treatment procedures, standards, and policies for such pollutants in the region.

**Supplementary Information:**

The online version contains supplementary material available at 10.1007/s11356-021-16558-8.

## Introduction

Over the past 30 years, factors such as population growth, urbanization, and intensification of agriculture have substantially increased water abstraction, use and pollution in the MENA region. The most water-scarce in the world, the region houses 6% of the global population with just 1% of the Earth’s renewable freshwater resources. The average availability of renewable water resources per capita in most of the MENA countries is less than 1000 m^3^/inhabitant/year, a threshold set by the Food and Agriculture Organization (FAO) and often used as an indicator of scarcity (Mualla 2018). This has repercussions across various sectors of the economy (World Bank 2012), with the situation only deteriorating due to climate change (Waha et al. 2017).

The region produces about 19 km^3^ of municipal wastewater annually but has the capacity to treat only about 50% of it (AbuZeid et al. 2019; AQUASTAT 2020). Since a part of the installed capacity is either inoperational or ill-maintained, the portion of the wastewater receiving treatment is quite low, and goop part of it is discharged untreated into the environment. Pollution from industries and agriculture further exacerbates the problem. As a result, more than 17% and 14% of the water bodies in the region are heavily affected by pathogen and organic pollution respectively, posing a risk to ecosystems and humans where the water is used for drinking, bathing, or irrigation (UNEP 2016).

The challenges posed by the occurrence of, and the exposure to conventional pollutants such as nitrates or pathogens in the MENA region are relatively well-documented (Hamed et al. 2013; UNEP 2016). However, data on the occurrence of EPs such as antibiotics, pharmaceuticals, personal care products (PCPs), or hormones (Geissen et al. 2015; Belver et al. 2016) are still patchy and scattered.

Emerging pollutants or emerging contaminants are typically defined as synthetic or naturally occurring pollutants, with known or suspected adverse ecological and/or human health effects, which are currently not included in routine environmental monitoring programs nor regulated in the environment (Unesco.org; Peña-Guzmán et al. 2019; NORMAN network 2019). The NORMAN network lists more than 1036 such compounds as EPs (NORMAN network 2019).

EPs include (a) substances recently introduced into the environment; (b) compounds present in the environment for a long time but not established to be potentially dangerous to the ecosystem and/or humans earlier (Sousa et al. 2018); and (c) compounds released into the environment for a long time but not recognized until the development of new detection methods (Geissen et al. 2015; Belver et al. 2016).

There are numerous sources of EPs ranging from hospitals and industries to agriculture and households. A given EP could also have more than one source. For instance, human pharmaceuticals could enter the environment through blackwater from households, hospital effluents, and effluents from pharmaceutical industries; caffeine could come from graywater, blackwater, and hospital effluents, while phenols could come from industrial activities and graywater. Graywater is generated from household sources (water from kitchens, bathing, washing clothes, and handwashing), and includes all sources other than toilets (which is considered as blackwater) (Craddock et al. 2020).

EPs are known to occur widely in freshwater, sewer overflows, and wastewater in Europe, North America, Brazil, Australia, India, and China (Petrie et al. 2015; Hughes et al. 2013; Copetti et al. 2019). Toxicity tests conducted under controlled laboratory conditions for different types of EPs (Della Greca et al. 2007; López-Serna et al., 2013; Hughes et al. 2013; Sanchez and Egea 2018) suggest that many of these compounds are potentially very toxic to aquatic life as well as human health. While EPs are generally present in freshwater within the ng/L range, with the risk of acute toxicity considered to be low or negligible, there are nevertheless notable exceptions. For example, the antibiotic ciprofloxacin has been found in high concentrations (6.5 mg/L) in a lake located in Patancheru in India’s Telangana state (Hughes et al. 2013). Other EPs such as carbamazepine are persistent and tend to accumulate in the environment, increasing toxicity hazard. Additionally, despite significant knowledge gaps, research suggests that the presence of some compounds, at or close to the current levels detected in the environment, may lead to chronic health effects (Kümmerer 2009a; Kümmerer 2009b). EPs also typically appear as a complex mixture, which can often lead to undesirable synergistic effects (Brain et al. 2004). Compounding the problem is the development and spread of antibiotic-resistant bacteria (ARB) which has been linked to the occurrence of antimicrobials in water and wastewater, posing a major public health concern (Marti et al. 2013; Bengtsson-Palme et al. 2018; Corno et al. 2019).

The presence of EPs in water and wastewater in other regions with similar characteristics and the associated potential negative effects on human health and the environment thus underlines the need for a better understanding of their occurrence, sources, and fate in the MENA region.

This review outlines the current knowledge on the occurrence of different EPs in wastewater, environmental waters and drinking waters in different countries in the MENA region, and discusses their sources and potential exposure pathways, with a focus on irrigation. The removal efficiency of different EPs in the treatment plants implemented at a full-scale level in the same region is also evaluated.

## Methodology

### Search, inclusion criteria, and database structure

The initial bibliographic search analyzed 14,400 papers in the Scopus database in the period between 1976 and September 2020, encompassing the very first EP monitoring initiatives as well as studies conducted on EPs in the region.

Studies were examined step-by-step based firstly on their titles, then the abstracts and finally the full texts. Search terms were selected in a way to ensure that all potentially relevant articles are accessed. The search was undertaken from country-to-country to include all the 18 MENA countries (Algeria, Bahrain, Egypt, Iraq, Israel, Jordan, Kuwait, Lebanon, Libya, Morocco, Oman, Palestine, Qatar, Saudi Arabia, Syrian Arab Republic, Tunisia, United Arab Emirates, and Yemen) using the following search format for titles or abstracts: [(“Emerging pollutants” OR “Contaminants of Emerging Concern” OR “Emerging Contaminants” OR “pharmaceuticals” OR “hormones” OR “microplastics” OR “PAHs” OR “PCBs”) AND (“Country’s name”) AND (“drinking water” OR “wastewater” OR “groundwater” OR “surface water” OR “treated wastewater”)]. The initial search utilizing the titles resulted in the pre-selection of 4320 studies. Abstracts—the summary of the publications—were then used for studies selection. Eligible studies including relevant description in the abstracts are subject to full-text evaluation. Abstracts that did not focus on water or the countries assessesd in this review were excluded. After analyzing the abstracts, 546 studies were selected to examine the full text. The criterion for inclusion in the publications collection was that a study should have explicitly analyzed, detected, and quantified at least one EP in either wastewater, TWW, surface water, groundwater, and/or drinking water in one or more countries in the MENA region.

The search identified a total of 89 studies, published from 1976 to September 2020, involved in our final database. The extracted data were compiled in a Microsoft Excel spreadsheet. The data were classified by water type and EP group. Fourteen studies from our final database were used to calculate the EP removal efficiency—if it had not been calculated by the authors—for 79 compounds in wastewater treatment plants (WWTPs) in the MENA region.

### List and grouping of EPs considered in the review

This review classified EPs into different groups based on Sousa et al. (2018) and Teodosiu et al. (2018). These groups are food additives, pharmaceuticals, PCPs, hormones/steroids, pesticides, plasticizers, phenols, illicit drugs, and other organic compounds (that cannot be classified in previous groups). Since some of these groups are not mutually exclusive, we listed some of the compounds in more than one EP group to avoid ambiguity (Table 1).

**Table 1:** Sources and examples of emerging pollutants

**Sources of EPs**		**Examples of EPs**	
		**EP group**	**Compound**
**Households**	**Blackwater**	Human hormones	Estrone, estriol, testosterone, 17β-estradiol
		Human pharmaceuticals	Carbamazepine, caffeine, naproxen, atenolol, ibuprofen, gemfibrozil, diclofenac
		Illicit drugs	Cocaine, MDMA
	**Graywater**	Personal care products	Triclosan, diethyl-meta-toluamide (DEET), parabens,benzylparaben, butylparaben
		Phenols	‘Soft’ ionic surfactants
		Food additives	Caffeine, sucralose, acesulfame
**Agricultural practices**		Veterinary pharmaceuticals	Vancomycin, sulfamethoxazole, diclofenac
		Aquaculture pharmaceuticals	Octyltetracycline
		Pesticides	Atrazine, triazine, simazine, hexachlorocyclohexane (HCH), gamma-hexachlorocyclohexane (γ-HCH), dichlorodiphenyltrichloroethane(DDT), dichlorodiphenyldichloroethane (DDD), dichlorodiphenyldichloroethylene (DDE), dieldrin
		Animal hormones	Estradiol, estrone, estriol
**Industrial activities**		Other organic compounds (that cannot be classified in other groups)	Polycyclic aromatic hydrocarbons (PAHs), polychlorinated biphenyls (PCBs), chlorinated hydrocarbons, perfluoroalkyl and polyfluoroalkyl substances (PFASs), volatile organic compounds (butylbenzene, m-xylene … )
		Plasticizers	Bisphenol A, phthalates, phthalate esters
		Personal care products	Tonalide, galaxolide, DEET, benzophenone, oxybenzone, parabens,benzylparaben, butylparaben,methylparaben
		Phenols	‘Hard’ non-ionic surfactants (2,4-dinitrophenol, 2-nitrophenol, octylphenol, nonylphenol mono and diethoxylates, nonylphenol carboxylates, nonylphenolethoxycarboxylates, nonylphenolethoxylates)
**Hospital effluents**		Human pharmaceuticals	Carbamazepine, ibuprofen, paracetamol, atenolol, lidocaine, clarithromycin, nacetylsulfamethoxazol (NACS)

EPs are known for their risk to the environment and human health. An assessment of such risk factors is mostly based on the persistence, bioaccumulation, and toxicity of the EPs (Rachel and Enda 2015). Substances that are toxic present a greater hazard when they are both persistent and bioaccumulative (Arnot and Gobas 2006). Persistence, a widely used parameter for EP prioritization and hazard characterization, is expressed in hours, days, or years and refers to the time that it takes an EP in the soil to change half of its mass to a different form or compound (UNEP 2012). Data on the persistence of EPs in soil are available mostly for pharmaceutical and personal care products (PCPs) and pesticides (Boxall et al. 2012). Globally, there is a dearth of literature on regulations surrounding the presence of some EPs as well as pharmaceuticals in water (Virkutyte et al. 2010). Table SI1 depicts available thresholds for EPs in drinking water, surface water, and groundwater in some countries in and beyond MENA region, together with their persistence in the environment. Thresholds in drinking water for EPs (pesticides and benzo[a]pyrene) are available for Bahrain, Egypt, Iraq, Jordan, Kuwait, Lebanon, Oman, Palestine, Sudan, Syrian Arab Republicn Arab Republic (WHO 2006; Omani Standard No. 8/2012).

In MENA region, regulations and guidelines for water reuse in agriculture are available for some contries, namely Tunisia, Oman, Israel, Saudi Arabia, Kuwait, Jordan, Palestine and Egypt (Shoushtarian and Negahban-Azar 2020). None of these regulations or guidelines have included EPs for agricultural water reuse, except phenols (expressed as total phenol) where thresholds are set by Oman (0.001 mg/l for category A: vegetables and fruit likely to be eaten raw and within 2 weeks of any irrigation and 0.002 mg/l for category B: vegetables to be cooked or processed, fruit if no irrigation within 2 weeks of cropping fodder, cereal seed crops, pasture, no public access), Saudi Arabia (0.002 mg/l), and Kuwait (1 mg/l) (Shoushtarian and Negahban-Azar 2020).

## Results

### Data availability and data gaps

**Fig. 1:**
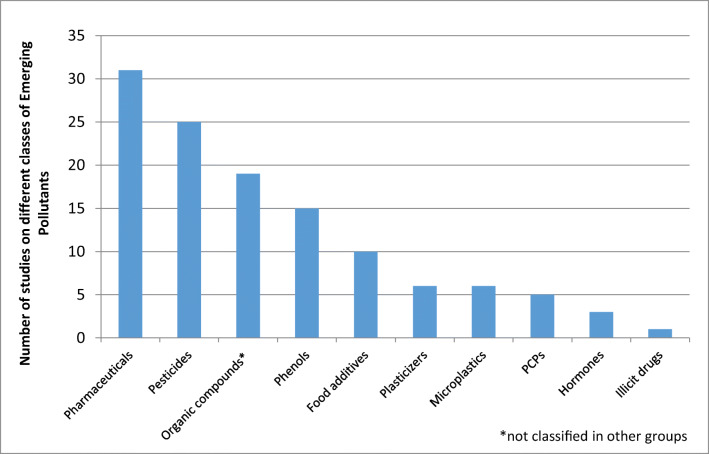
Number of publications dealing with the monitoring of different groups of EPs (from 1976 to September 2020) in wastewater, TWW, surface water, groundwater, and drinking waters in the MENA region

Our screening process resulted in 89 papers selected for our review. Figure 1 presents the number of studies reviewed by groups of EPs which shows that pharmaceuticals, pesticides, phenols, organic compounds, and food additives were the most frequently studied and reported compounds.

**Fig. 2:**
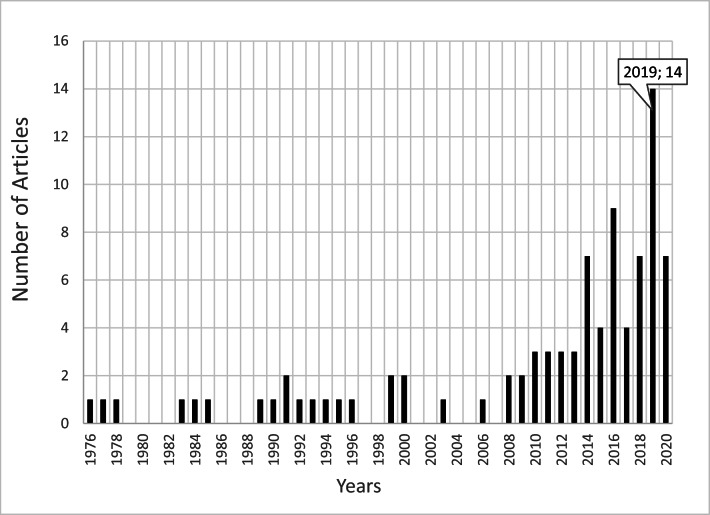
Year-wise distribution of 89 published studies drawn from a database (from 1976 to September 2020) dealing with the monitoring of EPs in wastewater, TWW, surface water, groundwater, and drinking waters in the MENA region

Between 1976 and 1998, there was scant research on the topic (Fig. 2), echoing a trend in other countries where this field of research was in its infancy. Since 2010, however, research in this field has gained great momentum globally, yet studies in the MENA region continue to be limited.

**Fig. 3:**
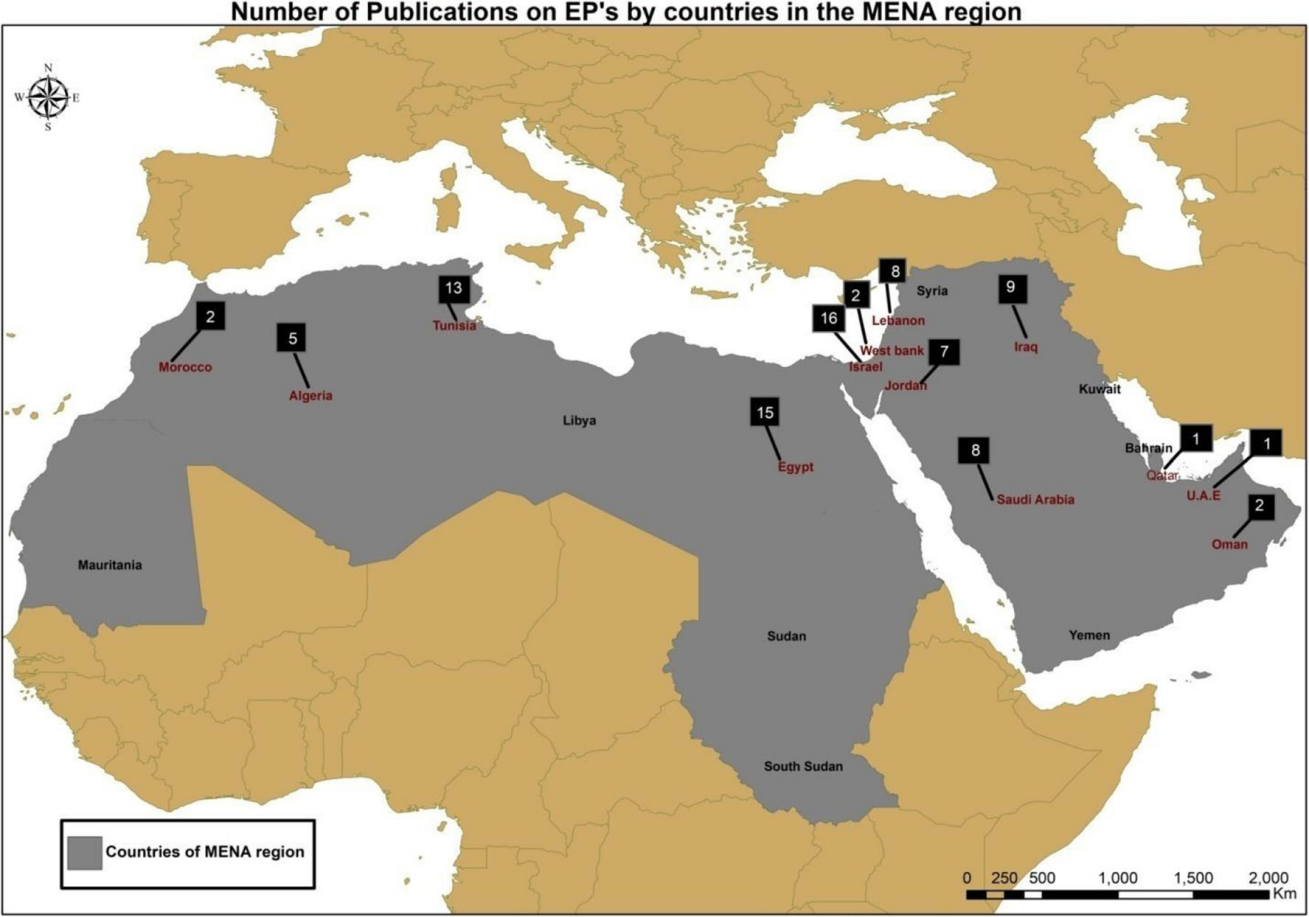
Distribution of studies on EPs in different water matrices by country in the MENA region

The distribution of studies on the occurrence of EPs in the water matrix of countries in the MENA region is presented in Fig. 3. Egypt, Israel, and Tunisia registered the highest number of studies. A lack or absence of data was observed for Sudan, Libya, Mauritania, Yemen, and Bahrain. Notably, this review is limited by a paucity or lack of studies in the majority of MENA countries, in part due to the lack of sensitive, selective, precise, and automatic methods (Lorenzo et al. 2018) to analyze the EPs. Published studies on EPs within the environmental matrix in this region are largely part of international projects funded and/or partnered by the EU, the USA, or other entities.

In “EPs occurrence in different water types,” we offer data on the concentration levels of EPs in different water matrices using box and whisker plots showing median values for the whole MENA region, as well as the variation (quartile groups) of the data. Due to data limitations, these plots present data derived from individual samples taken at different times and sites in the region using various extraction and/or analytical methods; therefore, the results need to be interpreted with caution. Medians and variations are disclosed to give an idea of the typical values and distribution for EPs in water. The relevant cases are then discussed in the text with emphasis on sources and concentrations of specific EPs.

### EPs occurrence in different water types

The subsections below illustrate the occurrence of EPs in wastewater, environmental waters, and drinking waters, wherein the concentrations, main sources, and the removal efficiency of EPs in implemented WWTPs in the MENA region are presented.

#### EPs in raw wastewater

**Fig. 4:**
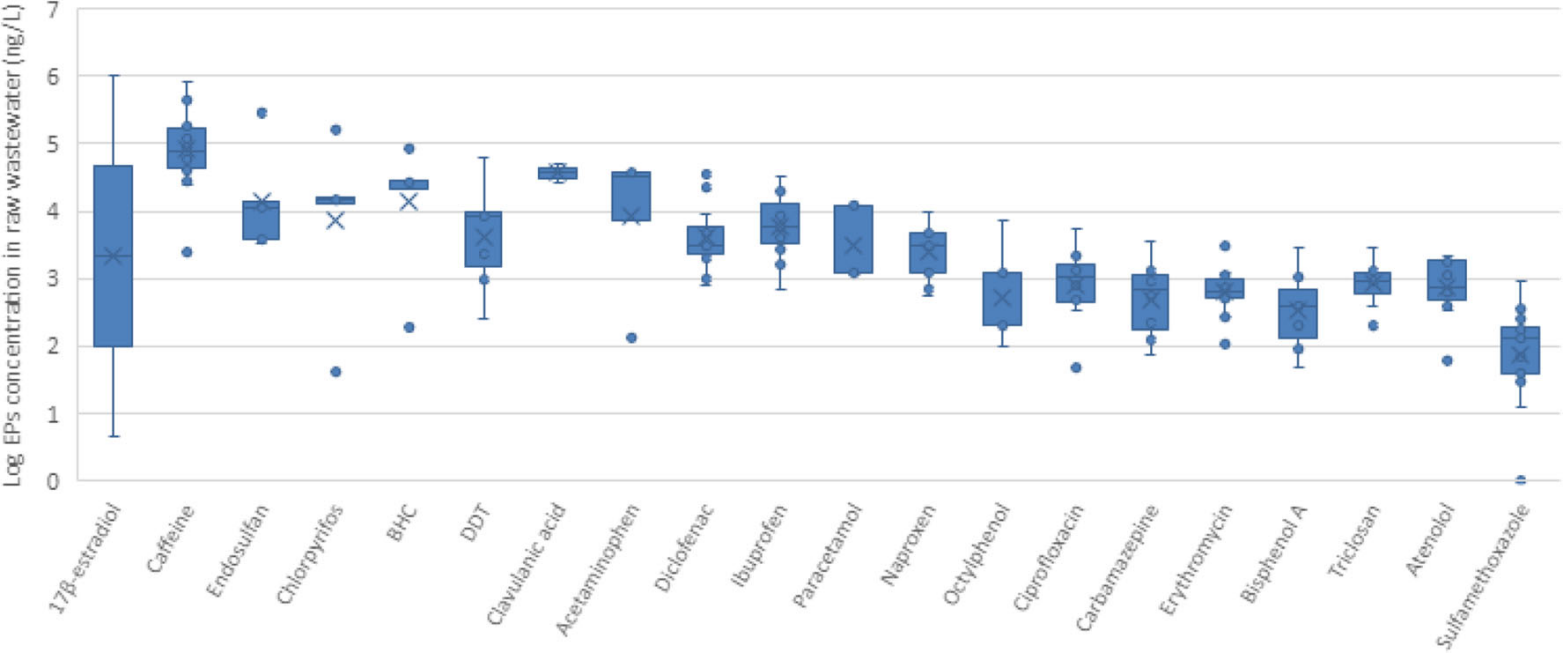
Median values and variation in the concentration levels of the 20 most-investigated EPs in individual samples of raw wastewater in the MENA region (*n*= 186)

Data on EPs in raw wastewater is given in Supplementary Table SI2. A total of 99 EPs, including one food additive, 57 types of pharmaceuticals, eight types of PCPs, four types of hormones, 25 types of pesticides, and one type of plasticizer and two types of phenols, were investigated in raw wastewater. Concentrations of the 20 most-studied EPs in individual samples of raw wastewater are given in Fig. 4.

Eight pharmaceutical compounds were among the 20 most frequently studied EPs in raw wastewater. Of these, the highest concentrations were observed for clavulanic acid (26,620–51,460 ng/L), diclofenac (800–35,333 ng/L), ibuprofen (700–34,000 ng/L), carbamazepine (73–3600 ng/L), erythromycin (1.5–3010 ng/L), atenolol (62–2198 ng/L), and sulfamethoxazole (12.1–900 ng/L) (Alahmad and Alawi 2010; Gasser et al. 2010; Al-Tarawneh et al. 2014; Al Qarni et al. 2016; Fries et al. 2016; Moslah et al. 2017; Al-Mashaqbeh et al. 2018; Al-Mashaqbeh et al. 2019b; Al-Maadheed et al. 2019; Craddock et al. 2020).

Caffeine has been detected in graywater and hospital and WWTP influents. A high concentration was found in influent samples taken from the As-Samra WWTP (182,500 ng/L) (Al-Mashaqbeh et al. 2018). A concentration of 1,076,000 ng/L was observed in the Baqa’a WWTP in Jordan (Alahmad and Alawi 2010).

There is greater interest in controlling pesticides in surface and groundwater than in raw wastewater as they are more likely to be exposed to such pollutants from agriculture. However, the presence of high levels of pesticides in raw wastewater is nevertheless worrying. The highest concentrations observed were for malathion (466,000 ng/L), DDT (2,300–61,000 ng/L), aldicarb (8900–42,400 ng/L), carbaryl (19,800–48,300 ng/L), chlorpyrifos (41.53–164,150 ng/L), endosulfan (190–290,200 ng/L), and pentachlorophenol (18,200–1,308,000 ng/L).

Natural hormones, like estriol, estrone, and testosterone were detected in raw wastewater with concentrations ranging from 18 to 360 ng/L, 42 to 152 ng/L, and 8 to 21.2 ng/L, respectively. 17β-estradiol (E2) is as well detected with concentrations ranging from 2.8 to 1,029,000 ng/L (Elnewishy et al. 2012; Dotan et al. 2016). The highest concentration of E2 (1,029,000 ng/L) was observed in the Bahr el-Baqar drain system in Egypt (Elnewishy et al. 2012).

In the case of PCPs, benzylparaben, butylparaben, ethylparaben, propylparaben, and methylparaben were observed in three coastal WWTPs in Tunisia. Concentrations ranged from 300 to 560,000 ng/L for propylparaben and methylparaben respectively (Hassine et al. 2011). Triclosan was detected in concentrations ranging from 200 to 2,800 ng/L in raw wastewater in Palestine and Israel. The lowest concentration was detected in graywater in Palestine while the highest concentration in influents was found in six WWTPs in Israel (Dotan et al. 2016). 1-H-benzotriazole and tolyltriazoles, used as corrosion inhibitors, have been detected in wastewater influents in concentrations of 65,500 ng/L and 10,400 ng/L respectively (Fries et al. 2016).

#### EPs in treated wastewater

**Fig. 5:**
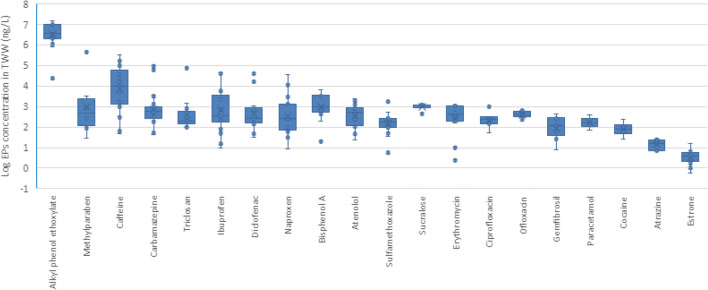
Median values and variation in the concentration of the 20 most-investigated EPs in individual samples of treated wastewater in the MENA region (*n*= 240)

According to our bibliographic database, 118 EPs were detected in TWW derived from WWTPs in the MENA region, including three types of food additives, 80 types of pharmaceuticals, five types of PCPs, three types of hormones, 14 types of pesticides, two organic compounds, eight types of plasticizers, two phenols, and one illicit drug (see Fig. 5 and Supplementary Table SI3 for more details).

Caffeine has been detected in TWW in MENA countries including Saudi Arabia, Jordan, Palestine, Israel, and Tunisia. Nineteen information points are available on caffeine in TWW in the MENA region, with concentrations ranging from 45.5 to 346,000 ng/L (Alidina et al. 2014; Alahmad and Alawi, 2010; Malchi et al. 2014; Fries et al. 2016; Moslah et al. 2017; Al-Mashaqbeh et al. 2019b; Craddock et al. 2020). The highest levels of caffeine were recorded in the Jordanian WWTPs, with an average concentration of 155,600 ng/L detected in the country’s largest treatment plant, As-Samra WWTP. The As-Samra TWW effluents are discharged into the King Talal dam which provides irrigation water for most of the agricultural activities in the Jordan Valley. A high concentration of caffeine (346,000 ng/L) was also reported in the Baqa’a WWTP in Amman (Alahmad and Alawi 2010).

Pharmaceuticals were also detected in TWW, with carbamazepine (concentration range 41–17,000 ng/L), atenolol (23.5–2380 ng/L), diclofenac (70–40,000 ng/L), erythromycin (2.4–1187 ng/L), ibuprofen (<10–40,000 ng/L), naproxen(2.94–1300 ng/L), and sulfamethoxazole (5.5–517.5 ng/L) counting as the most-investigated compounds in the MENA region (Avisar et al. 2009; Alahmad and Alawi, 2010; Alidina et al. 2014; Malchi et al. 2014; Al-Tarawneh et al. 2014; Zemann et al. 2014; Zemann et al. 2015; Al Qarni et al. 2016; Fries et al. 2016; Moslah et al. 2017; Al-Maadheed et al. 2019; Al-Mashaqbeh et al. 2019b; Khazri et al. 2019; Picó et al. 2019; Craddock et al. 2020). Significantly, TWW in most cases is reused for irrigation (of green areas, farms, trees n public parks, or fields) or groundwater replenishment. The highest concentrations of pharmaceuticals in TWW were reported for atenolol, carbamazepine, and sulfamethoxazole in Saudi Arabia; carbamazepine, diclofenac, ibuprofen, and naproxen in Jordan; and erythromycin in Tunisia (Table SI3). The variation could be linked to varying levels of consumption across the countries coupled with the properties of these compounds and the treatment processes adopted.

PCPs like parabens and triclosan too were detected in TWW. Parabens can originate in different EP groups as well as PCPs, pharmaceuticals, and food additives (Alan 2008). Certain metabolites of parabens are toxic to plant growth and development (Cecchi et al. 2004). The few studies investigating these pollutants in the MENA countries found methlyparaben concentrations to be ranging from 40 to 443,000 ng/L and propylparaben concentrations ranging from<20 to 585 ng/L in Saudi Arabia and Tunisia. The highest levels of methlyparaben were recorded in Tunisia and of propylparaben in Saudi Arabia (Hassine et al. 2011; Alidina et al. 2014; Fries et al. 2016).

Triclosan is an important antibacterial compound that is commonly used in hand soaps and industrial products such as toothpaste and antiseptic wipes (Daniel et al. 2017). It is a toxic compound characterized by its potential to create antibiotic resistance in bacteria (Gao et al. 2015). Given the same, triclosan was banned in the USA in September 2017 (Daniel et al. 2017); a ban is also being contemplated in Europe. It was observed in TWW in Saudi Arabia (Alidina et al. 2014), in Palestine and Israel (Dotan et al. 2016) with concentrations ranging from 100 to 74,000 ng/L. The highest level of the compound was detected in effluents in TWW from the WWTP in El Beireh (Palestine).

Bisphenol A was observed in TWW with concentrations ranging from <20 to 6679 ng/L. A maximum concentration of 6679 ng/L was observed in secondary effluents in the Wadi Al-Araj WWTP in Taif, Saudi Arabia (Al-Saleh et al. 2016). In the same study, high levels of bisphenol A were also reported in tertiary effluents, with concentrations of 3853 ng/L and 3628 ng/L recorded in the Manfouha and Wadi Hanifa WWTP effluents respectively (Al-Saleh et al. 2016).

The phenomenon of water reuse or discharge of completely treated wastewater allows any remaining compounds to enter the environment, especially when those compounds are not restricted/banned and are still in use. This TWW is used for different purposes, including irrigation of crops and green areas, groundwater recharge, or even indirect potable reuse in many countries across MENA (Alahmad and Alawi, 2010; Fries et al. 2016; Moslah et al. 2017; Al-Mashaqbeh et al. 2019b; Craddock et al. 2020). Nevertheless, there are several potential environmental and health-related risks associated with this practice. The use of TWW in irrigation may increase expand ARB and antibiotic-resistant genes levels in the soil (Gatica and Cytryn 2013).

#### EPs in surface water

EPs are capable of reaching surface water via many different pathways: agricultural activities, treated or untreated municipal wastewater, industrial effluents, human excretion, and domestic activities (Moreno-Gonzalez et al. 2013).

**Fig. 6:**
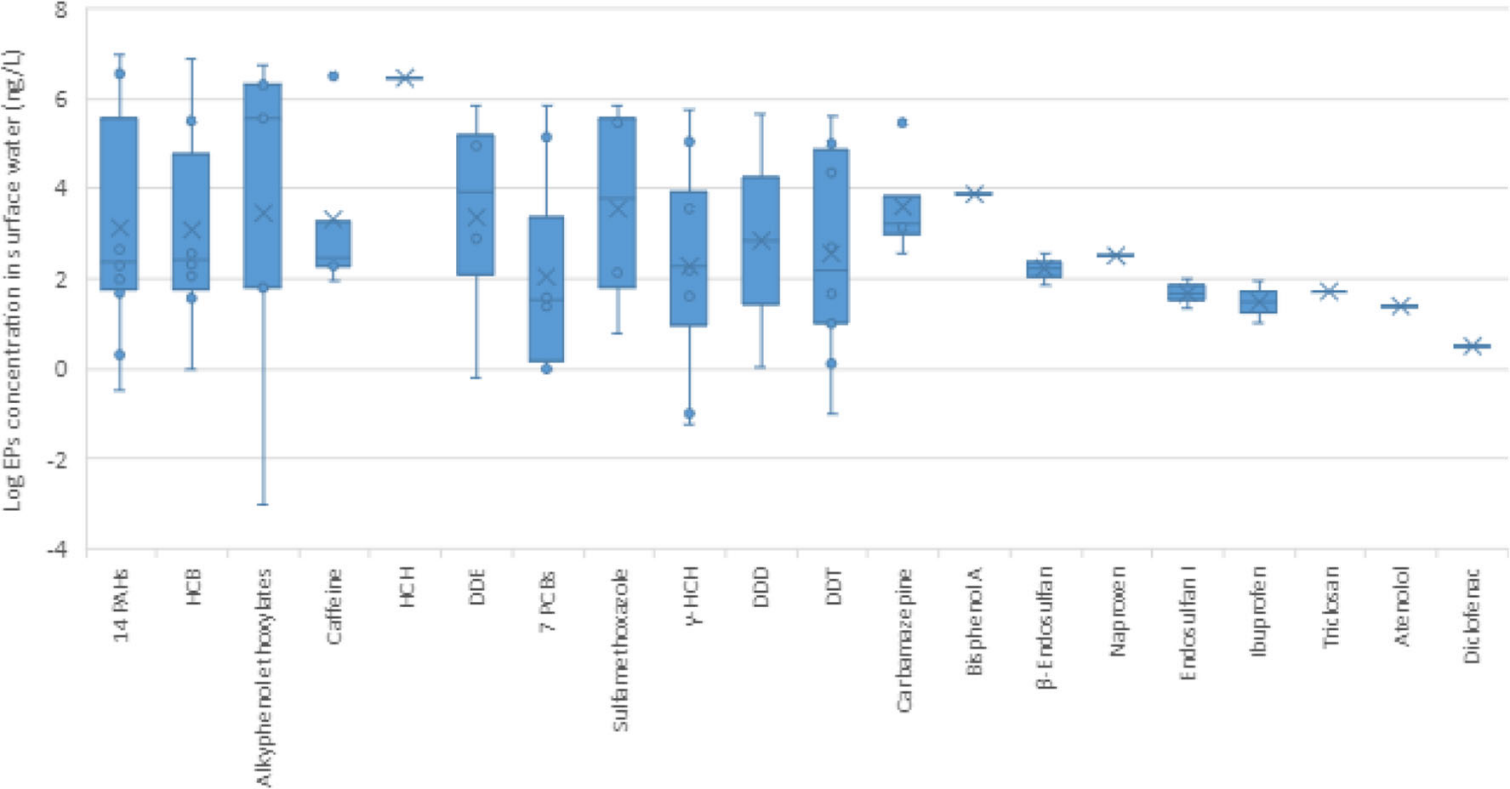
Median values and variation in the concentration levels of 20 most-investigated EPs in individual samples of surface water in the MENA region (*n*= 87)

According to our bibliographic database, 84 different EPs were found in surface water in the MENA region, including 32 types of pharmaceuticals, 30 types of pesticides, one food additives, 5 PCPs, one plasticizer, two phenols, one microplastic, and 12 types of other organic compounds. The concentration levels of the 20 most-investigated EPs in individual samples of surface water are given in Fig. 6. The data is presented in detail in Supplementary Table SI4.

While the EPs in Fig. 6 have mean values already exceeding safety limits for surface water, unsafe cases of high EP concentrations are also reported for other pollutants.

Walli (2015) reported an extreme case of industrial pollution of 53.7 mg/L for the sum of 13 priority PAHs in surface water samples, at the Al-Dalmaj marsh in Iraq’s Al-Diwaniya province. A high concentration (9.91 mg/L) was also reported for the sum of 14 priority PAHs in the El Bey river water in Tunisia (Khadhar et al. 2018). A later investigation in the same river by Gdara et al. (2018) revealed that industrial effluents contributed three times more to the contamination by PAHs of the river than agricultural effluents.

Fandi et al. (2009) found that the water discharged from the King Talal dam in Jordan was highly polluted with phenols (a sum of 65 compounds including cyclohexane and benzene), with an average concentration of 2.09 mg/L at the outlet of the dam and 1.82 mg/L in the reservoir. The sum of the so-called indicator PCBs registered the highest concentration of 0.47 mg/L in the El Bey river in Tunisia (Khadhar et al. 2018). These concentrations of phenols and PAHs far exceeded the safety limits set for surface water by the European Union (European Commission, Council Directive 2013/39/EC) (Table SI1). According to the previously cited studies, wastewater is a major contributor to surface water contamination by phenols, PAHs, and PCBs (Fandi et al. 2009; Khadhar et al. 2018; Gdara et al. 2018); a low degree of elimination of these compounds from WWTPs is responsible of their presence after treatment and consequently their dissemination and accidental pollution in the receiving environment.

Our dataset also revealed the presence of caffeine in different surface water bodies (Buerge et al. 2003); it was found in dam water in Jordan and in river water in Lebanon and Tunisia with a maximum concentration of 23,000 ng/L recorded in the Meliane river in Tunisia (Fries et al. 2016; Mokh et al. 2017; Al-Mashaqbeh et al. 2019a). The presence of caffeine typically indicates the strong influence of domestic wastewater on the quality of surface water (Viviano et al. 2017), since caffeine is widely used in a variety of foods (chocolate), beverages (coffee and tea), and drugs. While this study classifies caffeine under food additives, it could also emanate from pharmaceuticals.

A range of pharmaceutical compounds was also detected in surface water, of which carbamazepine is one of the most studied. It is characterized by its high persistence in diverse environmental matrices due to its low sorption and biodegradability (Bahlmann et al. 2014; Thelusmond et al. 2018; Zhang et al. 2008). Carbamazepine registered the highest concentration in the King Talal dam in Jordan (7500 ng/L) (Batarseh et al. 2013), exceeding the safety limit of 1000 ng/L set for the aquatic environment by the US-FDA. A later study by Al-Mashaqbeh et al. (2019a) reported a concentration of 358 ng/L in the same dam. Since the King Talal dam receives treated wastewater (TWW) from the As-Samra WWTP, the water quality may have improved after the plant was upgraded and expanded in 2015 (Al-Mashaqbeh et al. 2019b).

Pesticides too have been detected in surface water. The major pesticides observed were hexachlorobenzene (HCB), gamma-hexachlorocyclohexane (γ-HCH), dichlorodiphenyltrichloroethane (DDT), and dichlorodiphenyldichloroethylene (DDE). DDT was detected in numerous river waters used for irrigation and drinking in Lebanon (Badr et al. 2014; Youssef et al. 2015). Similarly, despite being banned in Egypt in 1980, DDT was detected in the country’s surface water in 2011 (El Bouraie et al. 2011). β-Endosulfan, a toxic pesticide, registered concentrations ranging from 24.46 to 55.32 ng/L—exceeding the probable effect level (PEL) of 3 ng/L—in three rivers that supply drinking and irrigation water in Lebanon (Badr et al. 2014; Youssef et al. 2015). In fact, at this PEL (set by the Canadian Council of Ministers of the Environment), adverse effects have been observed in 50% of marine environments (Helou et al. 2019). The agricultural activities around surface water sources have been reported to be the main sources of pesticides. Indeed, the massive and uncontrolled use of pesticides observed in some MENA countries like Lebanon only aggravates the situation (Helou et al. 2019).

Bisphenol A (BPA) is a plasticizer commonly used as a stabilizer and antioxidant in the production of plastics and food packaging (Chen et al. 2016). Human toxicity and ecotoxicity have been observed in the case of this compound over the past several years (Rochester 2013). As a result, Canada and the EU have prohibited the use of BPA in infant feeding bottles since 2011 (European Commission 2011). The American states of New York, Washington, and Minnesota have also strictly banned the use of BPA in products (Selvaraj et al. 2014). However, it is among the most well-documented chemicals reported in surface waters, although such studies in the MENA region are rare. BPA was detected in the Meliane River in Tunisia with a mean concentration of 784 ng/L. Its major sources are wastewater effluents and direct discharge of untreated wastewater into surface water (Fries et al. 2016). Aquatic predicted no-effect concentration (PNEC) values for BPA are available for several organisms like crustaceans, rotifers, insects, fish, algae, and aquatic plants. Selvaraj et al. (2014) reported BPA_PNEC_ values of 100 ng/L and 10 ng/L for mollusks and insect larvae, respectively. Levels of BPA reported in the Meliane river far exceed these BPA_PNEC_ values, and pose a threat to mollusks and insect larvae. Where river waters are used for irrigation and drinking purposes in the MENA region, constant monitoring of BPA should be considered.

#### EPs in groundwater

**Fig. 7:**
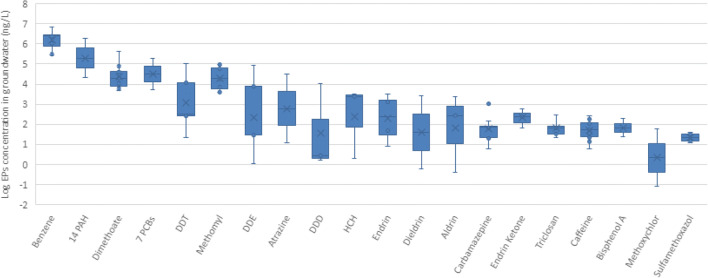
Median values and variation in concentration levels of 20 most-investigated EPs observed in individual samples of groundwater in the MENA region (*n*= 97)

Typically, groundwater is used as a source of drinking and/or irrigation water in the MENA countries. Ninety-seven different EPs were observed in groundwater in the region, including one food additive, 12 types of pharmaceuticals, four types of PCPs, 39 types of pesticides, one plasticizer, 11 types of phenols, and 29 types of other organic compounds. Detailed information is presented in Supplementary Table SI5. The concentration levels of the 20 most-investigated EPs in the individual samples of groundwater are shown in Fig. 7.

Twelve such pesticides are listed in the 20 most frequently researched EPs in groundwater in the MENA region. The highest concentration was reported for dimethoate (418,000 ng/L) in groundwater samples used for drinking purpose in Saudi Arabia (El-Saied et al. 2011), far exceeding the threshold set by the European Commission, regulation of 98/83/EEC for drinking water supply. By and large, as per health standards for drinking water, permissible pesticide levels are considered to be 100 ng/L (minimum concentration level, MCL) in the case of a single compound and 500 ng/L for the total concentration of organo-chlorinated pesticides. In marked contrast, a high concentration (11,840 ng/L) of DDD (a metabolite of DDT) was reported in groundwater samples used as well for drinking purpose from Akkar province, Lebanon, outstripping the maximum limit of 100 ng/L set by the European Commission directive 98/83/EEC for individual pesticides. The province is considered a major hotspot of pesticide use in Lebanon, with agricultural practices being the main source of groundwater contamination (Helou et al. 2019). Chbib et al. (2017) estimated that about 10.7 kg/ha of pesticides were used in this zone. Since people use this groundwater for both irrigation and drinking purposes (Helou et al. 2019), they are at risk of contamination by the pesticide residues. Although agriculture is an important source of pesticide contamination, pesticide manufacturing too can cause localized spikes in concentrations. In fact, in the Nir Galim area of Israel, groundwater near a pesticide plant was found to be contaminated by four pesticides (atrazine, alachlor, prometryn, and bromacil), with an average concentration of 31,250 ng/L, 204,500 ng/L, 37,750 ng/L, and 60,750 ng/L, respectively (Muszkat et al. 1993).

Organic compounds like PAHs and PCBs rank as well among the top 20 most-examined EPs in groundwater. Khadhar et al. (2018) studied their occurrence in 12 wells in the Grombalia aquifer in Tunisia and reported concentrations ranging from 20,400 to 1,930,000 ng/L for the sum of 14 priority PAHs and from 5200 to 196,000 ng/L for PCBs. Samples collected close to industrial areas and wastewater discharge locations were observed to have the highest concentrations of PAHs and PCBs. The concentrations recorded in this case for PAHs far exceeded the limits set for surface water (European Commission, Council Directive 2013/39/EC).

The practice of reusing TWW in agriculture (Elgala et al. 2003; Avisar et al. 2009; Gasser et al. 2010; Fries et al. 2016; Craddock et al. 2020) and aquifer recharge (Fries et al. 2016) also contributes to the accumulation of pharmaceutical compounds such as carbamazepine, clofibric acid sulfamethoxazole, and triclocarban in groundwater. In a recovery well in Israel, carbamazepine registered a concentration (1045 ng/L) surpassing the limit set by the US-FDA of 1000 (Gasser et al. 2010), while a lower concentration of 152 ng/L was detected in groundwater used for irrigation in Tunisia (Fries et al. 2016). Sulfamethoxazole was found in an area which had been irrigated by TWW for more than five decades, where monitoring wells and irrigation wells registered sulfamethoxazole concentrations of 32 ng/L and 12.5 ng/L respectively (Avisar et al. 2009). In Egypt’s El Gabal El Asfar region, clofibric acid concentration levels ranged from 40 to 75 ng/L in groundwater where raw wastewater has been used for more than 80 years (Elgala et al. 2003). Triclocarban too was detected in groundwater samples in the Jericho Governorate (Palestine) with a concentration level of 47.2 ng/L. Off-grid graywater treatment systems are in use in the studied zone; hence, the treated graywater used for irrigation could be the potential source of groundwater contamination with pharmaceutical compounds (Craddock et al. 2020).

#### EPs in drinking water

Access to safe drinking water is a vital social and environmental requirement. When drinking water does undergo contamination by EPs, the main agents are surface and groundwater pollution. Despite the importance of drinking water control, a study of the phenomenon in the MENA region first appeared as late as 2010 and even then dealt only with the presence of PAHs in drinking water in Iraq (Mohammed et al. 2010). Very few works are dealing with the occurrence of EPs in drinking water in the MENA region. Nine EPs were reported in drinking water (tap water and bottled drinking water) in the region, including four pharmaceuticals (ibuprofen, ketoprofen, ciprofloxacin, levofloxacin), three PCPs (methylparaben, propylparaben, butylparaben), one plasticizer (BPA), and one organic compound (PAH). The EPs investigated in individual samples of drinking water are given in Supplementary Table SI6 along with their concentration.

Finished drinking water samples from drinking water treatment plants in Algeria and Iraq were tested for pharmaceutical compounds by Kermia et al. (2016) and Mahmood et al. (2019). Their analysis revealed the presence of ibuprofen, ketoprofen and ciprofloxacin in concentrations ranging from 273 to 1312 ng/L.

Ciprofloxacin registered the highest concentration, of 1312 ng/L, in the drinking water used for human consumption (Mahmood et al. 2019), exceeding the maximum concentration of ciprofloxacin reviewed in our database for groundwater (23.8 ng/L), surface water (1058 ng/L), and even TWW (987 ng/l).

Sixteen priority PAHs were found in drinking water in Iraq in concentrations varying from 8.46 to 3654.8 ng/L (Jazza et al. 2016; Mohammed et al. 2010). BPA was detected in drinking water in Egypt and Saudi Arabia with concentrations ranging between 290 and 41,190 ng/L (Radwan et al. 2020; Alammari et al. 2020), much greater than the levels registered in surface water in the MENA region and worldwide. Given their possible direct impact on human health, these levels of BPA are worrying. PCPs as well as methylparaben, propylparaben, and butylparaben were also detected in high concentrations in drinking water in Egypt, with the highest concentration (6380 ng/L) being observed for butylparaben (Radwan et al. 2020).

#### Removal efficiency of EPs in WWTPs in the MENA region

Scientific studies offering the current satatus for EPs removal at WWTPs implemented in the MENA region were collected. The information on the percentage of removal using secondary, tertiary, and extensive systems is provided as supplementary information (Table SI7). The removal efficiency of the most-studied EPs—caffeine, carbamazepine, ciprofloxacin, diclofenac, erythromycin, estriol, estrone, and sulfamethoxazole—in existing WWTPs using varying treatments and technologies are given in Table 2. The removal efficiencies presented in Table SI7 and Table 2 are derived or calculated from the collected data, using concentrations of investigated EPs before and after treatment using the following formula:

$$ \% removal\ efficiency=\frac{\left[\mathrm{Influent}\right]-\left[\mathrm{Effluent}\right]}{\left[\mathrm{Influent}\right]}\times 100\% $$

**Table 2:** The removal efficiency of selected EPs in existing wastewater treatment plants in the MENA region.

	**Technology used**	**Removal efficiency (%)**							
		**CAF**	**CAR**	**CIPRO**	**DIC**	**ERY**	**E3**	**E1**	**SMX**
**WWT Technology group**									
Maximum secondary	Activated sludge	65	-40 to 22.5	55–100	-173.7 to 30.3	-25	97.6–100	74.6–98.8	-31 to 45.8
	Tricking filter	-	75	-	-	60–75			-
	Sequential biological reactor (SBR)	-		-	-	-	-	93.5	-
	Rotating biological contactor (RBC)	25	-43 to 60	-	44–100	20–100	-	-	#
	Aeration pond	-	-	-	-	-	-	93.2	-
Maximum tertiary	Polishing with sand filtration and disinfection with chlorine	>99	>86	26–>99		86.1–90.9	-	97.1* 94.7**	>98
	Membrane bioreactor (MBR)	-	-	-	-	-	-	98	-
	Aerated lagoon	-	-78	-	-	-	-	-	-
	Waste stabilization pond	-	0–33	-	65–100	17–100	-	-	100
Extensive systems	Soil-aquifer treatment	-	-	-	-	-	98–100	98–100	-

^#^Sulfamethoxazole was not detected in influent WWTPs but was found in the effluent in WWTPs (300 ng/L) using RBC treatment technology

*Polishing with sand filtration/**disinfecting with UV instead of chlorine/*CAF*, caffeine; *CAR*, carbamazepine; *CIPRO*, ciprofloxacin; *DIC*, diclofenac; *ERY*, erythromycin; *E3*, estriol; *E1*, estrone; and *SMX*, sulfamethoxazole

The chief secondary treatment processes used in WWTPs in the MENA region are activated sludge (AS), tricking filter (TF), sequential biological reactor (SBR), rotating biological contactor (RBC), aeration pond (AP), off-grid graywater treatment systems (off-grid GWTS), etc. Tertiary levels are offered with membrane bioreactor (MBR), aerated lagoon (AL), waste stabilization pond (WSP), reverse osmosis (RO), ultra-violet radiation (UV), ultrafiltration (UF), polishing with sand filtration (SF), disinfection with chlorine, etc. Soil-aquifer treatment (SAT) is seldom used as an extensive system.

Data on the EP removal efficiency of WWTPs is available for 79 compounds, with their concentrations in influent and effluent wastewater being distributed as follows: 61 pharmaceuticals, three hormones, three pesticides, six food additives, four illicit drugs, one phenol, and finally, one plasticizer.

For caffeine, secondary treatment was found to have a removal efficiency ranging from 25 to 69% (Craddock et al. 2020; Moslah et al. 2017; Alahmad and Alawi, 2010). Tertiary treatment, however, with SF and disinfection with chlorine was reported to remove up to 99% of the caffeine in wastewater (Al Qarni et al. 2016 and Al-Mashaqbeh et al. 2019b).

In many WWTPs across the MENA area, the removal efficiency for carbamazepine was shown to be negative (i.e., the pollutant’s concentration was higher in the effluent than in the influent). This was observed in seven WWTPs in Tunisia, where removal efficiencies were −40% in six plants that utilized AS process and −78% in the seventh WWTP which deployed AS/AL (Moslah et al. 2017). Negative removal (−43%) was also observed in the Al-Salt WWTP in Jordan which used RBC with extended aeration as treatment processes (Al-Tarawneh et al. 2014). Low removal of carbamazepine was also observed in different WWTPs in Jordan; in the old As-Samra WWTP (0% with AS/WSP), the new As-Samra WWTP (22.5% with AS/extended aeration), Abu-Nsair WWTP (27% with RBC/extended aeration), and Al-Aqaba WWTP (33% with AS/WSP) (Al-Tarawneh et al. 2014; Al-Mashaqbeh et al. 2019b).

Higher removal rates of carbamazepine of 60% and 75% respectively, were recorded in WWTPs applying RBC such as the Irbid WWTP and TF as in the Al-Karak WWTP in Jordan (Al-Tarawneh et al. 2014). The same deduction is valid for carbamazepine elimination in WWTPs using AS process, where different removal efficiencies were recorded by Moslah et al. (2017) (−78% and −40%), Al-Tarawneh et al. (2014) (0%), and Al-Mashaqbeh et al. 2019b (22.5%). The low removal rates for carbamazepine may be linked to the low water solubility, low sorption and low biodegradability of the compound, and by consequence its high persistence in diverse environmental matrices and high resistance to treatment (Bahlmann et al. 2014; Thelusmond et al. 2018; Zhang et al. 2008)

Ciprofloxacin removal efficiency was studied in two WWTPs in Doha which received the same flow rate of 54,000 m^3^/day and utilized similar technologies comprising of AS/SF and disinfection with chlorine as tertiary treatment. However, the removal efficiency of ciprofloxacin was found to be varying, with up to 70.5% removal with the old WWTP, but only 22% removal achieved with the new plant (Al-Maadheed et al. 2019). This could be due to dissimilarity in the influent origin, where the new WWTP received a major part of domestic and hospital wastewater while the old WWTP was limited to receiving tankers of septic tanks. Since influent wastewater characteristics differ (molecular weight, biodegradation, natural or anthropic origin, hydrophobicity, etc.), removal of different EPs using the same process consequently vary greatly, fluctuating TWW quality and the risk posed by water reuse.

A removal level of more than 99% was registered for ciprofloxacin using AS/SF and disinfection with chlorine in Saudi Arabia (Al Qarni et al. 2016) and using AS technology in Tunisia (Moslah et al. 2017). However, Harrabi et al. 2018 registered lower removal levels (55%) for ciprofloxacin in a WWTP in Sfax, Tunisia using the AS process. The latter WWTPs, recording lower ciprofloxacin removal, receives higher daily flow rate (60,000 m^3^/day) and higher pollution load (population equivalent of 526.800) compared to the WWTP studied by Moslah et al. (2017) that receives a daily flow rate (49,500 m^3^/day) with a population equivalent of only 195,000. Ciprofloxacin persists in activated sludge samples since it is a non-volatile compound (Batt et al. 2007) but is susceptible to photochemical degradation (Jelic et al. 2012). However, large amounts of organic matter in activated sludge may block the sunlight and therefore dampen the photochemical degradation (Kulkarni et al. 2017). These possible conditions could explain the lower removal efficiency of ciprofloxacin in the WWTP of Sfax. The treatment processes, the capacity of a WWTP to match the pollutant heaviness coupled with the pollutant characteristics, especially biodegradability, are therefore key factors to reach satisfactory removal rates of EPs.

Activated sludge seems to have been ineffective in the removal of diclofenac, with a removal efficiency of −174% to 30.3% reported in WWTPs in Algeria (Kermia et al. 2016). In Jordan, secondary treatment (RBC and TF) was observed to remove diclofenac by 44–100%. According to Alahmad and Alawi (2010) and Al-Tarawneh et al. (2014), the use of RBC led to more than 93% elimination of diclofenac in Abu-Nusair, Baqa’a and Al-Salt WWTPs, except for the Wadi Alseir WWTP in Jordan, where the efficiency was 44%. The TF process seems to be markedly less effective, with the diclofenac removal rates being 66% in Al-Karak WWTP and 75% in Irbid WWTP. In addition, the WSP too was found to be unsuitable for diclofenac removal—only a 65% removal efficiency was seen in the As-Samra WWTP (Al-Tarawneh et al. 2014).

Erythromycin registered a negative removal rate (−25%) using the AS treatment in Tunisia (Moslah et al. 2017). WSP as well was observed to achieve a very low removal of the compound (17%), while the TF technology proved to more promising, with removal rates ranging from 60 to 75%. However, a huge variation in the removal rate (20–100%) was observed using RBC (Al-Tarawneh et al. 2014). The best removal results of 86.1–90.0% were obtained with tertiary treatment (SF/UV/Chlorine or SF/ Chlorine) (Al-Maadheed et al. 2019).

Secondary treatment is considered to be adequate for both estriol and estrone removal. The removal rates ranged from 74.6 to 100% with secondary treatment (SBR, AS/bottom aeration, AS/rotating disks) and reached more than 94.7% with tertiary treatment (SF, SF/UV, MBR). SAT was shown to achieve removal rates of 98–100% for both estriol and estrone (Dotan et al. 2016).

Our bibliographic database revealed low removal success rates for SMX in the MENA region using secondary treatment (AS, off-grid GWTS, WSP). Removal efficiency did not exceed 45.8%; indeed, even negative removal (−31%) was registered (Al-Tarawneh et al. 2014; Moslah et al. 2017; Al-Mashaqbeh et al. 2019b). Other studies in the literature have also observed negative removal efficiency for SMX (Bendz et al. 2005; Sim et al. 2010; Tewari et al. 2013). More serious negative removal rates for SMX were reported as -145.6% in China (Zhang et al. 2017), −107% in Switzerland (Göbel et al. 2007) and −133.4% in Turkey (Nas et al. 2021). According to Nas et al. (2021), negative removal efficiency for SMX is observed especially during the summer period. Furthermore, SMX, while being absent in the WWTP influent, was detected in the effluent in Al-Salt WWTP in Jordan, which used RBC with extended aeration as the treatment process (Al-Tarawneh et al. 2014). This could be due to an accumulation of micro-contaminants in biological sludge without real degradation and their transmission by far (Jelic et al. 2011). Tertiary treatment has the potential to improve the removal efficiency of SMX. Both SF/Chlorine and WSP have proven effective in removing more than 98% of SMX residues (Al-Tarawneh et al. 2014; Al Qarni et al. 2016).

## Discussion

### Monitoring and capacities

According to our database, 290 EPs have been reported all over the MENA region in raw wastewater, TWW, surface water, groundwater, and drinking water; nevertheless, the distribution in time and space of these data is quite heterogeneous and patchy. There are no consistent monitoring programs at the national or the regional MENA level which allow for the development of time series for the analysis of trends for a given EP in a given water type of water body. The published studies are mostly part of international projects funded and/or partnered by western donors which result only in partial snapshots. The paucity of data in the majority of MENA countries is chiefly driven by the lack of proper legislation and capacities. The monitoring of EPs is costly and requires knowledge and skills and sophisticated analytical equipment, protocols, and procedures to analyze EPs, by being able to detect pollutants at a very low concentration in different water matrices (Lorenzo et al. 2018). Furthermore, proper monitoring requires the capacity to store, process, and interpret data to inform policy and practice through evidence. Many MENA countries do not yet have these capacities, nor the regulations that make monitoring compulsory. Recently, Al-Maadheed et al. (2019) attributed the lack of analytical studies on the presence of EPs in wastewaters to social (absence of policies and/or regulations) and technical factors (measuring and reporting on such contaminants) within the Gulf Cooperation Council; the same phenomenon is valid for additional water matrices like drinking water (Mohammed et al. 2010), in other such countries in the region.

Due to the lack of consistent monitoring, reported data on EPs remains limited. Nevertheless, the scientific community and general public are increasingly becoming more aware of the presence of EPs in drinking water and the aquatic environment, along with the associated risks (Hendry, 2017). This increase in knowledge is driving a change, with the number of studies dealing with EPs in water increasing, as visible as a trend in Fig. 2.

### Occurrence of EPs in wastewater and removal efficiency in WWTPs

Although some EPs (like caffeine) may be naturally present in environmental waters, the occurrence of EPs is mainly due to increasing pollution loads from anthropic sources such as cities, agricultural activities, and industries. Wastewater, livestock waste, and agricultural drainage lead to a concentration of residues from pharmaceuticals, pesticides, microplastics, PCPs, and other EPs. As shown in our database, raw municipal wastewater in the MENA region has been reported to concentrate pesticides like endosulfan or DDT, pharmaceuticals such as acetaminophen, ibuprofen, paracetamol, naproxen, diclofenac, or carbamazepine, and other pollutants. The limited actual treatment of these wastes and wastewater in many MENA countries results in a large portion of these EPs making their way to water bodies, in turn increasing the risk of exposure downstream.

Even in the cases where wastewater is collected and treated, the removal efficiency for EP in existing WWTPs is at best limited. Existing secondary treatments are effective for some compounds like E1 and E3 but ineffective for others like CAF, CAR, CIPRO, DIC, ERY, and SMX. Activated sludge in particular, which is the most commonly used technology in MENA, is inadequate in its effectiveness in the removal of most EPs. CAR, ERY, and SMX appear to be the hardest compounds to eliminate utilizing existing secondary treatments; at times, even negative removals were observed. The negative removal of pharmaceutical compounds could be a result of the persistence (Gulkowska et al. 2008), recombination, and accumulation of compounds during secondary treatment (Kagle et al. 2009; Jelic et al. 2011; Verlicchi et al. 2012; Gao et al. 2016).

Better removal rates are achieved with tertiary treatment for certain EPs, but the performance of different technologies is very inconsistent and heterogeneous. As a result, it is difficult to find cost-effective combinations of technologies that work well for all or most of the EPs. For instance, WSP is not suitable for ERY and CAR elimination. SF/UV/chlorine is more suitable for ERY and CAR but is not suitable for CIP. SF/chlorine has also been shown to be effective for ERY—the process can almost completely degrade this contaminant (86.1–90.9%)—however, the same process is less effective for some compounds like amoxicillin (46.3–45.1%) and CIPRO (26–70.5%) and only negligibly effective for Tetracycline (0–19.2%) (Al-Maadheed et al. 2019).

The reported removal efficiency for the same compounds by the same combination of technologies is at times different. We speculate that these differences and the negative removal cases for EPs removal during treatment may be due to (i) the type of influent wastewater containing pollutants which may inhibit the effectiveness of biological treatment or favor transformation, recombination, degradation and/or accumulation of EPs; (ii) treatment conditions (e.g., climatic conditions, or the age of the treatment plant) that can affect the performance of WWTPs; and (iii) the specific practices or operational conditions (e.g., shorter retention times when the treatment plant is overloaded and overloading the plant beyond its capacity with excess flows).

**Fig. 8:**
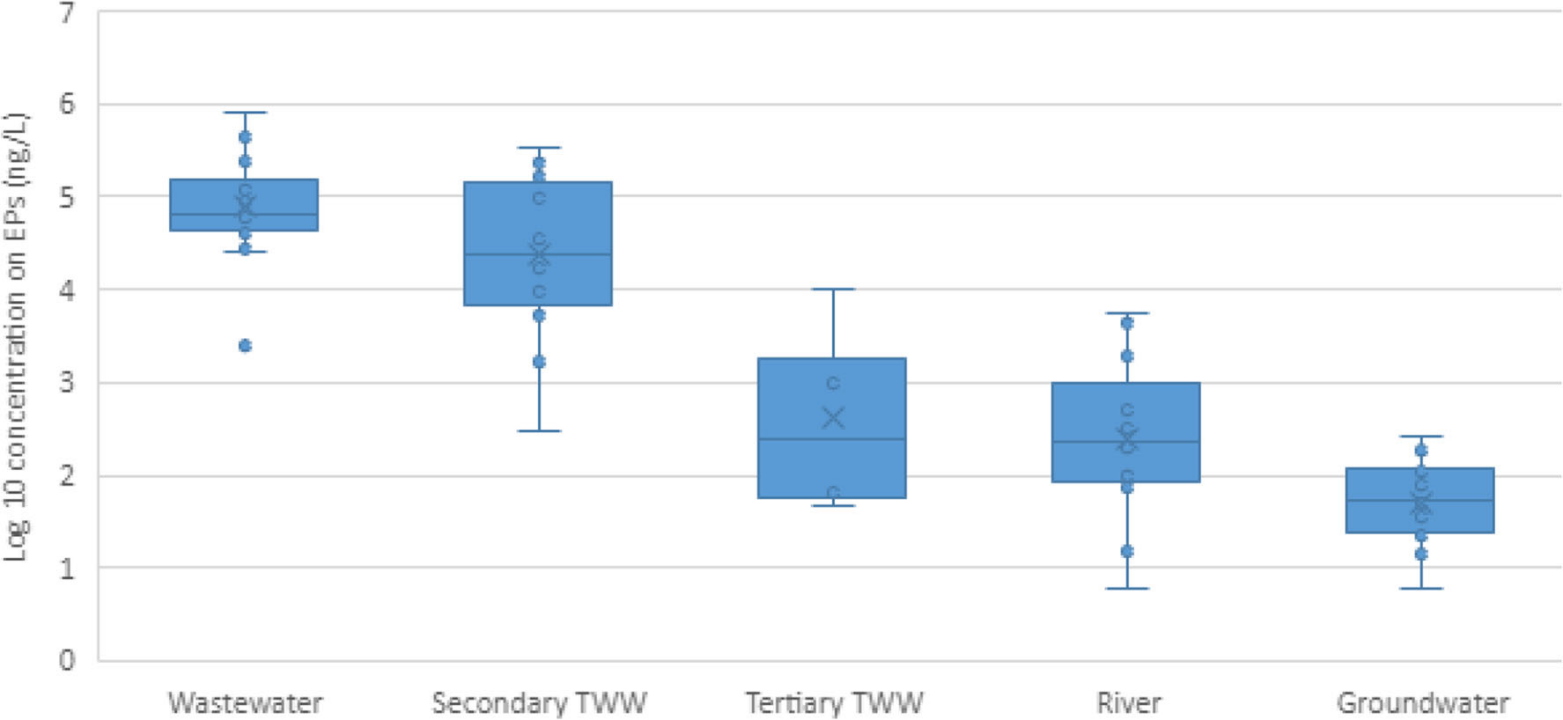
Box and whisker plot showing the median value and variation in caffeine concentrations in individual samples taken from different water types (*n*= 59) in the MENA region. (Mokh et al. 2017; Craddock et al. 2020; Moslah et al. 2017; Hassine et al. 2011; Alahmad and Alawi, 2010; Al Qarni et al. 2016; Fries et al. 2016; Alidina et al. 2014; Malchi et al. 2014)

Caffeine is a case in point. The most-studied waterborne EP, there are a total of 59 data points for caffeine concentration in different types of water in the MENA region. Figure 8 shows the aggregated data for different water types across the MENA. The available data allows us to infer that while the existing secondary treatment shows little removal efficiency, the tertiary treatment’s effectiveness averages a 2 log reduction in the caffeine concentration. Concentration in tertiary effluents is similar to the concentration reported in surface waters across MENA.

### Exposures pathways and risks

EPs from deficiently treated or non-treated waste and wastewater contribute to polluting the receiving waters, which may have significant health implications for water users downstream. Humans may be exposed to these waterborne EPs, by drinking polluted water or by using polluted water in food production, processing, and preparation.

MENA is one of the most water-scarce areas in the world; as a response to this, it is also a region with many projects facilitating the direct use of treated wastewater in irrigation. Jordan, Israel, and Tunisia are considered advanced in TWW reuse in the MENA region. For instance, in Jordan, about 95% of wastewater is treated and more than 92% of it is reused in agricultural activities (Al-Mashaqbeh et al. 2019b). Nevertheless, even in these countries, the quality of the reused water is very heterogeneous and in most cases, the existing treatments have limited removal capacities to treat EPs. There are also numerous informal instances of direct use of untreated wastewater in agriculture where farmers take wastewater directly from sewerage systems to irrigate. The most common practice, however, is the indirect reuse of untreated or partially treated wastewater, wherein the wastewater is released into surface water, diluted, and subsequently reused, e.g., in agriculture. A typical regional example of this practice is the irrigation along the Nile and the Nile delta. There is increasing evidence that pharmaceutical compounds, PAHs, PCBs, and pesticides can accumulate in TWW irrigated soil, as illustrated in the case of Nabeul, Tunisia, where water reuse has been prevalent in agriculture for more than 30 years (Fenet et al. 2012; Haddaoui et al. 2016). The long-term health effects of EPs in sites where wastewater (treated or not) is used directly or indirectly in agriculture are still not well-documented.

Sewage sludge, which is a by-product of wastewater treatment, tends to accumulate the EPs and ends up becoming a pathway to the dispersion of the EPs in the environment when such sewage sludge is reused in agriculture.

Surface and groundwater receive the EPs not only from municipal wastewater but also through agriculture (e.g., pesticides), livestock waste (e.g., antibiotics), and industrial wastewater (e.g., pharmaceuticals from the pharma industry). Groundwater contamination with EPs may have long-term and hard-to-revert impacts (Neshat and Pradhan, 2015). The long residence time of EPs in certain types of aquifers, like fossil aquifers or confined aquifers where pollution persists for tens or hundreds of years, can make water unsafe for extensive periods of time. If we consider the use of many such aquifers for drinking purposes, the issue becomes even more worrying and calls for strong precautionary measures to protect the aquifers in the first place.

In spite of the same, according to our database, 57 cases of surface water, groundwater, and drinking water have been reported to have concentrations of EPs that exceed the tolerable limits summurized in table SI1, out of wchich 52 cases are reported after 2010: carbamazepine with four cases in surface water in Jordan; ∑PAHs with 14 cases in surface water in Algeria, Jordan, Iraq, and Tunisia, two cases in groundwater in Saudi Arabia and Tunisia and two in drinking water in Iraq; endrin with one case in groundwater in Lebanon; aldrin with one case in surface water in Lebanon; dieldrin with one case in surface water in Lebanon; lindane with one case in surface water used for drinking purpose in Egypt; dimethoate with 12 cases in groundwater in Saudi Arabia; DDD with one case in groundwater in Lebanon; DDT with five cases in surface water in Egypt, three in groundwater in Lebanon and Saudi Arabia, heptachlor three cases in surface water in Egypt, one case in groundwater in Lebanon, heptachlor epoxide two cases in surface water in Egypt, two cases in groundwater in Lebanon; and β-endosulfan with two cases in surface water in Lebanon. Such levels are disturbing, if this water is used in irrigation of crops to be eaten raw, and indeed, dangerous in the case of water bodies used for bathing and drinking water.

### Implications for policy and practice

While our results show an increasing number of studies lately on water-borne EPs in the MENA region, it is important to keep raising awareness about the occurrence of potential long-term risks associated with uncontrolled EPs disposal in the environment. As the MENA countries become increasingly aware of the issue and start transitioning to a more comprehensive set of water quality norms that include EPs, they will need to undertake priority setting, select EP indicators based on defined criteria such as occurrence and exposure, persistence, bioaccumulation, and toxicity along with conducting environmental and health risk assessments (Mansour et al. 2016; Semerjian et al. 2018). The countries should then progressively build their capacities to robustly monitor these priority EPs. This includes not only the development of accredited and well-equipped labs but also building the capacity to store, process and interpret data to inform policies and practices.

The WWT technologies that have been considered effective for traditional pollutants such as COD, nitrogen, or pathogens have not proven effective in removing several EPs in MENA, these findings are in agreement with worldwide studies (Zhang et al. 2017; Anderson et al. 2010; Nas et al. 2021). A selection of more effective technologies is required for the better elimination of EPs and achieving safer water reuse in the region. Advanced oxidation processes (AOPs) and advanced reduction processes (ARPs) appear to be promising in reducing organic compounds, pharmaceuticals, CECs, PCPs, EDCs, and pathogens. The combination of AOPs and ARPs in advance oxidation and reduction processes (AORPs) allows overcoming some limits for certain compounds like dichlorophenol, some PFAS or perchlorate (Capodaglio 2020). Carbamazepine is among the pollutants which need to be considered well for removal improvement during treatment in WWTPs in the region, using more suitable processes. Tertiary treatment technology using a combination of SF and disinfection with chlorine demonstrates a removal efficiency of more than 86% (Al Qarni et al. 2016).

Wastewater treatment is one option to address water pollution from EPs, but a combination of solutions works better than wastewater treatment alone. Pollution can be prevented at the source by not only effecting a change in consumption and production patterns (e.g., by reducing the use of pharmaceuticals, pesticides, or plastics) but also by improving agricultural drainage management (Zandaryaa and Mateo-Sagasta 2018; Mateo-Sagasta et al. 2018, Nikiema et al. 2020). Exposure to EPs can be managed through, for example, drinking water treatment in conjunction with adopting safe water practices in food production and processing since there is a potential formation of new compounds (e.g., disinfection by-products) by oxidation during treatment (Sirivedhin and Gray 2005; Qu et al. 2009).

## Conclusions and recommendations

Data on the EPs control in environmental, raw, and drinking waters are even now sparse and scattered. Many countries in the region have had no studies done on waterborne EPs. Nevertheles, despite the paucity of data, there is increasing evidence highlighting the presence of EPs in waters across the region. Two hundred and ninety EPs have been reported all over the MENA region, of which, 99 EPs are detected in raw wastewater, 118 in TWW, 83 in surface water, and 9 in drinking water. Pharmaceutical compounds are among the top reported compounds. In groundwater, 97 different types of EPs have been reported with pesticides as the most frequent ones. The occurrence of EPs in groundwater is particularly disquietening since the retention times of the pollutants in groundwater are larger than in other water bodies; even if pollution loads are arrested, the EPs may take years to be removed.

There is anecdotal evidence that the concentration of EPs has at times reached beyond safe limits. Fifty-seven cases of waters used, or potentially used, for drinking purposes have been reported to have a concentration of EPs that surpass the tolerable limits for drinking water.

Despite the available studies on EPs in water, the problem is nevertheless not fully characterized. Indeed, the reported occurrences may be only the tip of the iceberg. More studies are necessary for a stronger assessment of the contamination status of different types of water bodies.

Given the large portion of the generated wastewater in the MENA countries which is directly or indirectly reused in irrigation, the exposure pathways to EPs through the use of contaminated water in food production merits special attention.

Removal efficiency differs significantly by the treatment technology and type of EP. The existing data on EP removal effectiveness in the MENA countries suggest that secondary treatment is ineffective in the reduction of most EPs (e.g., pharmaceuticals compounds like carbamazepine, erythromycin, sulfamethoxazole). Tertiary treatment improves the elimination of many EPs, but this improvement is inadequate for some pollutants (e.g., tetracycline, ciprofloxacin, and amoxicillin). The construction and operation of tertiary treatment are costly processes; many MENA countries may face fiscal challenges in being able to implement such expensive treatments. The extent of the wastewater treatment coverage and the types of WWT and drinking water treatment technologies in the MENA region are far from sufficient to effectively address the health risks posed by the EPs. Given the limited financial capacities of the middle- and low-income MENA countries, and the limited effectiveness of EPs removal by the tertiary treatments, it is not practical nor affordable to promote wastewater coverage with treatment as the only way to address waterborne EPs. Instead, we recommend prioritizing a more cost-effective combination of solutions that includes a change in consumption and production patterns to prevent pollution at the source, wastewater treatment expansion to the extent required for conventional pollutants including pathogens, adoption of good irrigation practices, and universal coverage of drinking water treatment.

Since it is one of the first such comprehensive reviews of the sources, occurrence and removal efficiency of EPs in the MENA countries, this study could serve as baseline data to design future water resource monitoring programs in the region. Apart from addressing the data and geographical gaps, such future monitoring programs can focus their attention on the EPs which emerging evidence indicate to be posing higher health risks.

## Supplementary information

ESM 1 (DOCX 152 kb)

## Abbreviations

EPs: Emerging pollutants
MENA: Middle East and North Africa
TWW: Treated wastewater
WWTPs: Wastewater treatment plants
FAO: Food and Agriculture Organization of the United Nations
ARB: Antibiotic-resistant bacteria
PCPs: Personal care products
PAHs: Polycyclic aromatic hydrocarbons
PCBs: Polychlorinated biphenyls
DEET: Diethyl-meta-toluamide
HCH: Hexachlorocyclohexane
γ-HCH: Gamma-hexachlorocyclohexane
DDT: Dichlorodiphenyltrichloroethane
DDD: Dichlorodiphenyldichloroethane
DDE: Dichlorodiphenyldichloroethylene
E2: 17β-estradiol
EE2: 17α-ethynyle stradiol(EE2)
E3: Estriol
US-FDA: United States Food and Drug Administration
EU: European Union
EEC: European Community Directive
Dutch DWD: Dutch Drinking Water Directive
BPA: Bisphenol A
PEL: Probable effect level
PNEC: Predicted no-effect concentration
MCL: Minimum concentration level
AS: Activated sludge
TF: Tricking filter
SBR: Sequential biological reactor
RBC: Rotating biological contactor
AP: Aeration pond
GWTS: Graywater treatment systems
MBR: Membrane bioreactor
AL: Aerated lagoon
WSP: Waste stabilization pond
RO: Reverse osmosis
UV: Ultra-violet radiation
UF: Ultrafiltration
SF: Sand filtration
SAT: Soil-aquifer treatment
CAF: Caffeine
CAR: Carbamazepine
CIPRO: Ciprofloxacin
DIC: Diclofenac
ERY: Erythromycin
E3: Estriol
E1: Estrone
SMX: Sulfamethoxazole
