# Supplementary material for: A review on occurrence of emerging pollutants in waters of the MENA region
Source: Environ Sci Pollut Res Int. 2021 Oct 19;28(48):68090–110. doi: 10.1007/s11356-021-16558-8 (PMC8718386; doi:10.1007/s11356-021-16558-8)
Supplement: Supplementary file 1 — (DOCX 152 kb) [file 11356_2021_16558_MOESM1_ESM.docx]

Table SI1: Concentration limits of selected EPs in drinking water, surface water and groundwater and reported persistence in the environment as per selected sources.

| **Emerging pollutants (EPs)** | **Limit**  **(ng/L)** | **Regulation name and/or issuing agency** | **Persistence** | |
| --- | --- | --- | --- | --- |
|  |  |  | **Half-life in soil (days)** | **Half-life in water (days)** |
| **Drinking water** | | | | |
| **Pharmaceuticals** | | | | |
| Carbamazepine | 40,000  50,000  100,000  1,000* | Minnesota Department of Health  Australia  Dutch DWD (2011)  United States Food and Drug Administration (US-FDA)  United States Food and Drug Administration (US-FDA) | 46–>120 (Li et al. 2013)  125 ((2) Durán-Álvarez et al. 2012)^**^ | 63-1200 (Tixer et al. 2003 ; Zou et al. 2015) |
| Diclofenac | 7,500 | Dutch DWD (2011) | 8.47 (Xu and Chang 2009)  20.44 (Xu and Chang 2009)^**^ | 8–35 (Buser et al. 1998) |
| Clofibric acid | 30,000 | Dutch DWD (2011) | 56.4 (Chen et al. 2013) ^**^ | 11(Cardinal et al. 2016) |
| Ibuprofen | 150,000 | Dutch DWD (2011) | 6.09 (Xu and Chang 2009)  121.9 (Carr et al. 2010)^**^ | 4.6- 413(Fono et al. 2006 ; Yamamoto et al. 2009) |
| Sulfamethoxazole | 75,000 | Dutch DWD (2011) | 2 (Liu et al. 2010) ^**^ | 17(Cardinal et al. 2016) |
| **Personal care products** | | | |  |
| Triclosan | n.a. | - | 18 (Ying et al. 2007) | 4-8(Aranami and  Readman. 2007) |
| **Hormones** | | | |  |
| Steroid hormones:  Estrone(E1)  17β-estradiol(E2)  17α-ethynyle Stradiol(EE2)  Estriol(E3) | n.a. | - | 13.7 (Chen et al. 2013)  2.3 (Carr et al. 2010)  3.0 (Carr et al. 2010)  1.7 (Carr et al. 2010) | - |
| **Polychlorinated biphenyls** | | | |  |
| Individual PCBs | 100 | Dutch DWD (2011) | 210–330 (Gan and Berthouex 1994) | - |
| ∑PCBs | 500 | Dutch DWD (2011) and European Commission, Council Directive 80/778/EEC |  |  |
| **Phenols** | | | |  |
| Individual phenol  Sum phenol | 100  500 | European Commission, Council Directive 80/778/EEC |  |  |
| Surfactants | 200,000 | European Commission, Council Directive 80/778/EEC | 7–33 (Waters et al.1989; Berna et al. 1989) |  |
| Benzene | 1,000 | European Commission, Council Directive 98/83/EEC and Dutch DWD (2011) | 30^(12)^  0.5–10 (Howard 1991) |  |
| Benzene | 10,000 | WHO (2003) |  |  |
| Benzene | 10,000  70,000 | Jordarian Drinking-water Standards; JS 286:2001  Syrian Arab Republic Drinking-water Quality, Ministry of Environment/Higher Council for  Environment Safety, 1994  Decree of Minister of Health of Egypt No (108) and (301)/1995, Ministry of Health)  Omani Standard No. 8/2012 “Un- Bottled Drinking Water”  Drinking-water Standard ICS 13.060.00, Sudanese Standards and  Metrology Organization, 2002 |  |  |
| **Polycyclic aromatic hydrocarbons** | | | | |
| Benzo[a]pyrene | 10 | Dutch DWD (2011) | 151 days–14.6 years (Wild et al.1991; Keck et al. 1989) | 4 days (rivers and lakes) (Vozhzennikov et al., 1997)  80 days (coastal sea water) (Vozhzennikov et al., 1997)  1600 days (ocean water) (Vozhzennikov et al., 1997)  2 months (Mackay et al., 1992) |
| Benzo[a]pyrene | 700 | WHO (2003) |  |  |
| Benzo[a]pyrene | 700    500 | Decree of Minister of Health of Egypt No (108) and (301)/1995, Ministry of Health)  Omani Standard No. 8/2012 “Un- Bottled Drinking Water”  Drinking-water Standard ICS 13.060.00, Sudanese Standards and  Metrology Organization, 2002 |  |  |
| Individual PAHs  benzo[b]fluoranthene  Benzo[k]fluoranthene  Benzo[ghi]perylene  Indeno[1,2,3-cd]pyrene | 100 | European Commission, Council Directive 98/83/EEC | 87 days-14.2 years (Wild et al.1991; Keck et al. 1989)  139 days–11 years (Wild et al.1991; Symons et al. 1988)  173 days-1.8 years (Symons et al. 1988; Coover and Sims 1987)  58–730 ((Symons et al. 1988; Coover and Sims 1987) | 4 - 7 years (Howard et al., 1991 ; Bisson et al., 2005)  5 – 23 (Howard et al., 1991)  -  - |
| ∑PAHs | 100 | Dutch DWD (2011) and European Commission, Council Directive 98/83/EEC | - | - |
| **Pesticides** | | | | |
| Chlorpyrifos | 30,000 | WHO (2003) | 10–120 () Meikle and Hedlund 1973; Bidlack 1979) | - |
| Chlorpyrifos | 30,000 | Omani Standard No. 8/2012 “Un- Bottled Drinking Water” | - | - |
| Endrin | 600 | WHO (2004) | 333–4300 (Laskowski et al. 1983; Castro and Yoshida 1971) | 4300 (Rousseau et al. 2007) |
| Endrin | 600 | Omani Standard No. 8/2012 “Un- Bottled Drinking Water” | - | - |
| Aldrin  Dieldrin | 30  30 | WHO (2003) | Aldrin: 291 days-9 years (Nash and Woolson 1967; Lichtenstein and Schulz 1959)  Dieldrin: 175-1080 (Castro and Yoshida 1971; Kearney et al. 1969) | Aldrin: 365 (Rousseau et al. 2007)  Dieldrin: 1000 (Rousseau et al. 2007) |
| ∑ Aldrin and dieldrin | 30  20 | Jordarian Drinking-water Standards; JS 286:2001  Syrian Arab Republic Drinking-water Quality, Ministry of Environment/Higher Council for  Environment Safety, 1994  Decree of Minister of Health of Egypt No (108) and (301)/1995, Ministry of Health)  Ministry of Environment of Lebanon. Decree No. 52/1–Standards for the Minimization of  Pollution of Air, Water and Soil, 1996  Omani Standard No. 8/2012 “Un- Bottled Drinking Water”  Sudan Drinking-water Standard ICS 13.060.00, Sudanese Standards and Metrology Organization, 2002 | - | - |
| Lindane | 2,000 | WHO (2003) | 15 months (Wauchope et al. 1992) | 4.7 years in surface water (Strand and Hov, 1996)  28 years in deep water (Strand and Hov, 1996)  2 years (Mackay et al., 1991) |
| Lindane | 2,000  4,000  1,500 | Syrian Arab Republic Drinking-water Quality, Ministry of Environment/Higher Council for  Environment Safety, 1994  Decree of Minister of Health of Egypt No (108) and (301)/1995, Ministry of Health)  Jordarian Drinking-water Standards; JS 286:2001  Omani Standard No. 8/2012 “Un- Bottled Drinking Water”  Palestine Standard 41, Palestine Standards Institution, 1997  Sudan Drinking-water Standard ICS 13.060.00, Sudanese Standards and Metrology Organization, 2002 |  |  |
| Dimethoate | 6,000 | WHO (2004) | 4.8–9.7 (Kolbe et al. 1991) | 3,7 – 118 (U.S. National Library of medicine. 1995) |
| Dimethoate | 6,000 | Omani Standard No. 8/2012 “Un- Bottled Drinking Water” |  |  |
| DDD | 100 | WHO (2004) | >30 years (Nash and Woolson 1967) | >150 years (U.S. National Library of medicine. 1995) |
| DDT | 100 | WHO (2004) | 20–30 years (Nash and Woolson 1967) | >150 years (U.S. National Library of medicine. 1995) |
| ∑DDT and metabolites | 1,000 | WHO (2004) | - | - |
| ∑DDT and metabolites | 2,000  1,500  1,000 | Decree of Minister of Health of Egypt No (108) and (301)/1995, Ministry of Health)  Jordarian Drinking-water Standards; JS 286:2001  Sudan Drinking-water Standard ICS 13.060.00, Sudanese Standards and Metrology Organization, 2002  Syrian Arab Republic Drinking-water Quality, Ministry of Environment/Higher Council for  Environment Safety, 1994  Omani Standard No. 8/2012 “Un- Bottled Drinking Water” | - | - |
| Atrazine | 2,000 | WHO (2003) | Atrazine: 20–50 USEPA. 1988 | Atrazine: > 100 (Royal Society of Chemistry. 1991) |
| ∑Atrazine and its metabolites | 100,000 | WHO (2011) | - | - |
| Individual pesticides (aldrin, dieldrin, heptachlor and heptachlor epoxide) | 30 | European Commission, Council Directive 98/83/EEC | - | Heptachlor: 4 -5 (INERIS, 2005) |
| Individual pesticides | 100 | European Commission, Council Directive 98/83/EEC and Dutch DWD (2011) | - | - |
| Metolachlor | 10,000  10,000  7,000 | WHO (2004)  Decree of Minister of Health of Egypt No (108) and (301)/1995, Ministry of Health)  Omani Standard No. 8/2012 “Un- Bottled Drinking Water”  Drinking-water Standard ICS 13.060.00, Sudanese Standards and Metrology Organization, 2002 | 14 (Ettore. 2008) | 90-610 (Rousseau et al. 2007; Ettore. 2008) |
| Pentachlorophenol | 9000  7000  0.2 mg/l | Decree of Minister of Health of Egypt No (108) and (301)/1995, Ministry of Health)  Omani Standard No. 8/2012 “Un- Bottled Drinking Water”  Drinking-water Standard ICS 13.060.00, Sudanese Standards and  Metrology Organization, 2002 | 13 – 130 (IARC. 1991) | 48 (Rousseau et al. 2007) |
| Trichlorophenol | 0.15 mg/l  0.01 mg/l | Decree of Minister of Health of Egypt No (108) and (301)/1995, Ministry of Health)  Drinking-water Standard ICS 13.060.00, Sudanese Standards and  Metrology Organization, 2002  Syrian Arab Republic Drinking-water Quality, Ministry of Environment/Higher Council for  Environment Safety, 1994  Drinking-water Standard ICS 13.060.00, Sudanese Standards and  Metrology Organization, 2002 | 127 – 927 (HSDB) | 2.6 (Scully and Hoigne. 1987) |
| Chlorophenol 2 | 5 mg/L | Drinking-water Standard ICS 13.060.00, Sudanese Standards and  Metrology Organization, 2002 | 15 -23 (Ettinger and Ruchhoft. 1950) |  |
| Dichlorophenol, 2,4- | 2 mg/L | Drinking-water Standard ICS 13.060.00, Sudanese Standards and  Metrology Organization, 2002 | 15 – 312 (Smith and Aubin. 1991) | 2.6 – 13 (Lyman et al. 1982; Scully and Hoigne. 1987) |
| Trichlorophenol,2,4,6- | 10 mg/L | Syrian Arab Republic Drinking-water Quality, Ministry of Environment/Higher Council for  Environment Safety, 1994 | 8 – 14 (ToxNet. 2016) | 7 (Tabak et al. 1981) |
| Chlordane | 2,000  2,000  1,500 | WHO (2004)  Decree of Minister of Health of Egypt No (108) and (301)/1995, Ministry of Health)  Syrian Arab Republic Drinking-water Quality, Ministry of Environment/Higher Council for  Environment Safety, 1994  Omani Standard No. 8/2012 “Un- Bottled Drinking Water”  Sudan Drinking-water Standard ICS 13.060.00, Sudanese Standards and Metrology Organization, 2002 | 283 days to 3.8 years (surface water)  566 days to 7.6 years (groundwater)  (Howard 1991 in Franse and de Voogt, 1997) | 350 (Rousseau et al. 2007) |
| Atrazine | 2,000  2,000  1,500 | WHO (2004)  Decree of Minister of Health of Egypt No (108) and (301)/1995, Ministry of Health)  Ministry of Environment of Lebanon. Decree No. 52/1–Standards for the Minimization of  Pollution of Air, Water and Soil, 1996  Omani Standard No. 8/2012 “Un- Bottled Drinking Water”  Sudan Drinking-water Standard ICS 13.060.00, Sudanese Standards and Metrology Organization, 2002 | 40 - 750 (Greve. 1996) | 60 (Rousseau et al. 2007) |
| Alachlor | 2,000  20,000 | WHO (2004)  Decree of Minister of Health of Egypt No (108) and (301)/1995, Ministry of Health) | > 30 (RIWA. 1998) | 15 (Rousseau et al. 2007) |
| Aldicarb | 10,000  10,000  7,500 | WHO (2004)  Decree of Minister of Health of Egypt No (108) and (301)/1995, Ministry of Health)  Omani Standard No. 8/2012 “Un- Bottled Drinking Water”  Sudan Drinking-water Standard ICS 13.060.00, Sudanese Standards and Metrology Organization, 2002 | 170 (ECCO. 1997) | 30 (Rousseau et al. 2007) |
| ∑Pesticides | 500 | European Commission, Council Directive 98/83/EEC and Dutch DWD (2011) | - | - |
| **Surface water** | | | | |
| **Emerging pollutants (EPs)** | **Limit**  **(ng/L)** | **Regulation name and/or issuing agency** | | |
| **Personal care products** | | | | |
| Triclosan | 100 | European Commission, Council  Directive 2013/39/EC | | |
| **Phenols** | | | | |
| Phenol | 7700 | European Commission, Council  Directive 2013/39/EC | | |
| **Polycyclic aromatic hydrocarbons** | | | | |
| Benzo(a)pyrene | 27 | European Commission, Council  Directive 2013/39/EC | | |
| **Pesticides** | | | | |
| Alachlor | 700 | European Commission, Council  Directive 2013/39/EC | | |
| Benzene | 50000 |  |  |  |
| Chlorpyrifos | 100 |  |  |  |
| Cyclodiene pesticides: Aldrin, Dieldrin, Endrin, Isodrin | 5 |  |  |  |
| Dimethoate | 480 |  |  |  |
| DDT total | 25 |  |  |  |
| Endosulfan | 10 |  |  |  |
| Heptachlor and heptachlor epoxide | 3 |  |  |  |
| **Groundwater** | | | | |
| **Emerging pollutants (EPs)** | **Limit**  **(ng/L)** | **Regulation name and/or issuing agency** | | |
| **Pesticides** | | | | |
| Aldrin | 22.5 | European Commission, Council  Directive 2006/118/EC | | |
| Benzene | 750 |  |  |  |
| Pesticides  Individual  sum | 100  500 |  |  |  |

*: limit set for aquatic environment / ** =Cases where soils are irrigated with treated wastewater.

n.a.= not available

Informations (regulation name, issuing agency, year of issuance) of national Regulation for drinking water per MENA country are as follow: Palestine (Palestine Standard 41, Palestine Standards Institution, 1997)); Sudan (Drinking-water Standard ICS 13.060.00, Sudanese Standards and Metrology Organization, 2002); Syrian Arab Republic (Syrian Arab Republic Drinking-water Quality Standards, Ministry of Environment/Higher Council for Environment Safety, 1994); Tunisia (Project of Tunisian Standard PTS 14.09, National Institute of Standardization and Industrial Property, 1993); Bahrain (Decree No.(10) for Environmental Standards (water and air); Egypt (Decree of Minister of Health and Population No.(108),1995); Jordan (Drinking-water Standards; JS 286, 2001).

Table SI2: Concentration ranges, number of information points of each emerging pollutant detected in raw wastewater in MENA region

| **Total number/group** | **Detected pollutants** | **Min- Max Concentration (ng/L)** | **Number of information points** | **Country** |
| --- | --- | --- | --- | --- |
| **Food additives** |  |  |  |  |
| 1 | Caffeine | 2444-827000 | 16 | Tunisia, Jordan, Saudi Arabia, Palestine |
| **Pharmaceuticals** |  |  |  |  |
| 1 | Amphetamine | 129 | 1 | Jordan |
| 2 | Aparamycin | 700-1500 | 3 | Tunisia |
| 3 | Atenolol | 62-2198 | 11 | Tunisia, SaudiArabia |
| 4 | Azithromycin | 56.5 - 660 | 3 | Egypt, Palestine, Tunisia |
| 5 | Carbamazepine | 73-3600 | 18 | Tunisia, Saudi Arabia, Jordan, Israel |
| 6 | Cephalexin | 23.3 - 1880 | 2 | Saudi arabia, Tunisia |
| 7 | Chloramphenicol | 500-3300 | 2 | Tunisia |
| 8 | Ciprofloxacin | 47,8-5600 | 8 | Egypt, Saudi arabia, Qatar, Palestine, Tunisia |
| 9 | Clarithromycin | 38-124 | 5 | Tunisia, SaudiArabia |
| 10 | Clavulanic acid | 26620-51460 | 3 | Qatar |
| 11 | Cotinine | 59-4890 | 3 | Jordan |
| 12 | Diclofenac | 800-35333 | 12 | Jordan, Algeria |
| 13 | Dihydrostreptomycin | 900 | 1 | Tunisia |
| 14 | Diphenhydramine | < 5 | 1 | Jordan |
| 15 | Erythromycin | 1.5-3010 | 13 | Qatar, Tunisia, Jordan |
| 16 | Fenofibric acid | 1250.00 | 1 | Tunisia |
| 17 | Florfenicol | 900-3300 | 4 | Tunisia |
| 18 | Gentamycin c1 | 600-800 | 3 |  |
| 19 | Gentamycin c1a | 50014870 | 3 | Tunisia |
| 20 | Gentamycin c2 | 1000 | 1 | Tunisia |
| 21 | Glimepiride | 1000-63333 | 5 | Jordan |
| 22 | Ibuprofen | 700-34000 | 9 | Jordan, Tunisia, Algeria |
| 23 | Kanamycin B | 500-7500 | 4 | Tunisia |
| 24 | Lidocaine | 129-158 | 2 | SaudiArabia |
| 25 | Linezolid | 1.7 | 1 | Palestine |
| 26 | MDMA | 12 to 18 | 2 | Jordan |
| 27 | Metformin | 15200 | 1 | Saudiarabia |
| 28 | Methotrexate | 179000-580667 | 6 | Jordan |
| 29 | Metronidazole | 548-3130 | 3 | Qater |
| 30 | Morphine | 42-46 | 2 | Jordan |
| 31 | NACS (Nacetylsulfamethoxazol) | 506-1200 | 2 | SaudiArabia |
| 32 | naproxen | 700-5200 | 6 | Jordan, Algeria |
| 33 | Neomycin | 1800-16400 | 4 | Tunisia |
| 34 | norfloxacin | 226700 | 1 | Tunisia |
| 35 | Norfluoxetine | 7070 | 1 | Saudiarabia |
| 36 | Ofloxacin | 138-868 | 7 | Tunisia |
| 37 | O-NAP | 151.00 | 1 | Tunisia |
| 38 | Oxacilin | 59.9 | 1 | Palestine |
| 39 | Oxazepam | 32.00 | 1 | Tunisia |
| 40 | Oxolinic acid | 35.8 | 1 | Palestine |
| 41 | Paracetamol | 1155.00-12400 | 5 | Tunisia, SaudiArabia |
| 42 | Paromycin | 1200-4200 | 2 | Tunisia |
| 43 | Penicillin | 120-220 | 3 | Qatar |
| 44 | Penicillin G | 37.3 | 1 | Palestine |
| 45 | Phenazone | 37-42 | 2 | Jordan |
| 46 | Pipemidic acid | 61.4 | 1 | Palestine |
| 47 | Sisomycin | 2300-6700 | 3 | Tunisia |
| 48 | Spiramycin | 690.5 - 84200 | 2 | Tunisia |
| 49 | Streptomycin | 1600-2700 | 2 | Tunisia |
| 50 | Sulfamethazine | 21-69 | 2 | Jordan |
| 51 | Sulfamethoxazole | 12.1- 900 | 15 | Egypt, Tunisia, Jordan, Saudi Arabia, Palestine |
| 52 | Tetracycline | 210 | 4 | Qatar |
| 53 | Thiabendazole | 12 to 15 | 2 | Jordan |
| 54 | Thiamphenicol | 1200 | 1 | Tunisia |
| 55 | Triclocarban | 251.4 | 1 | Palestine |
| 56 | Trimethoprim | 24- 171 | 3 | Jordan |
| 57 | Vancomycin | 2.8 | 1 | Palestine |
| **Personal care products** |  |  |  |  |
| 1 | Benzylparaben | 1500-4000 | 3 | Tunisia |
| 2 | Buthylparaben | 400-16300 | 3 | Tunisia |
| 3 | Ethylparaben | 1000-2500 | 3 | Tunisia |
| 4 | Propylparaben | 300-2700 | 3 | Tunisia |
| 5 | Methylparaben | 1000-560000 | 3 | Tunisia |
| 6 | 1-H-benzotriazole (corrosion inhibitor) | 65,500 | 1 | Tunisia |
| 7 | Tolyltriazoles (corrosion inhibitor) | 10,400 | 1 | Tunisia |
| 8 | Triclosan | 200 - 2800 | 7 | Palestine, Israel |
| **Hormones** |  |  |  |  |
| 1 | 17β-estradiol | 2.8-1029000 | 3 | Egypt, Israel |
| 2 | Estriol | 18- 360 | 10 | Palestine, Israel, Tunisia |
| 3 | Estrone | 41.8-151.9 | 8 | Palestine, Israel |
| 4 | Testosterone | 8-21.2 | 8 | Palestine, Israel |
| **Pesticides** |  |  |  |  |
| 1 | malathion | 466000 | 1 | Egypt |
| 2 | p,p'-DDT | 950 | 1 | Egypt |
| 3 | profenofos | 41000 | 1 | Egypt |
| 4 | BHC | 190-86200 | 6 | Egypt |
| 5 | DDT | 2300-61000 | 5 | Egypt |
| 6 | Alachlor | 5 | 1 | Palestine |
| 7 | Aldicarb | 8900-42400 | 4 | Egypt |
| 8 | Atrazine | 9.8 | 1 | Palestine |
| 9 | Carbaryl | 19800-48300 | 4 | Egypt |
| 10 | Carbofuran | 12000-21000 | 3 | Egypt |
| 11 | Chlorpyrifos | 41.53-164150 | 5 | Egypt |
| 12 | chlorpyrifos-methyl | 21800 | 1 | Egypt |
| 13 | diazinon | 47550 | 1 | Egypt |
| 14 | Dimethoate | 12400 | 1 | Egypt |
| 15 | Drins= aldrin,endrin,dieldrin,pentachloroanisole and pentachlorobenzene | 37400-185600 | 5 | Egypt |
| 16 | Endosulfan | 190- 290200 | 5 | Egypt |
| 17 | Heptachlor | 190-12100 | 6 | Egypt |
| 18 | Heptachlor epoxid | 10700-27800 | 5 | Egypt |
| 19 | Lindane | 630 | 1 | Egypt |
| 20 | O,p' -DDT | 250 | 1 | Egypt |
| 21 | Parathion | 10800 | 1 | Egypt |
| 22 | pentachlorophenol | 18200-1308000 | 3 | Egypt |
| 23 | pirimiphos-methyl | 23300 | 1 | Egypt |
| 24 | prothiphos | 30.03 | 1 | Egypt |
| 25 | Trifluralin | 0.8 | 1 | Palestine |
| **Plasticizers** |  |  |  |  |
| 1 | Bisphenol A | 50-2910 | 7 | Palestine, Israel |
| **Phenols** |  |  |  |  |
| 1 | Octylphenol | 200-7200 | 5 | Israel |
| 2 | alkylphenolethoxylates | 7040 | 1 | Israel |

Table SI3: Concentration ranges, number of information points of each emerging pollutant detected in treated wastewater in MENA region

| **Total number/group** | **Detected pollutants** | **Min- Max Concentration (ng/L)** | **Number of information points** | **Country** |
| --- | --- | --- | --- | --- |
| **Food additives** |  |  |  |  |
| 1 | Acesulfame | 1430-3830 | 4 | Saudi Arabia |
| 2 | Caffeine | 45.5-346000 | 18 | Saudi Arabia, Jordan, Palestine, Israel, Tunisia |
| 3 | Sucralose | 445-1250 | 4 | Saudi Arabia |
| **Pharmaceuticals** |  |  |  |  |
| 1 | 1,7-Dimethylxanthine | 14 | 1 | Jordan |
| 2 | 2-OH-ibuprofene | 8170 | 1 | Tunisia |
| 3 | Acebutolol | 3.92-7.36 | 2 | Emirate |
| 4 | Acetaminophen | 41-31200 | 2 | Saudi Arabi, Jordan |
| 5 | Amikacin | 1000-1800 | 2 | Tunisia |
| 6 | Amitriptyline | 25.5-365 | 4 | Saudi Arabia |
| 7 | Amoxicillin | 60-70 | 2 | Qatar |
| 8 | Amphetamine | 26 | 1 | Jordan |
| 9 | Aparamycin | 400-500 | 2 | Tunisia |
| 10 | Atenolol | 23.5-2380 | 19 | Emirate, Tunisia, Saudi Arabia |
| 11 | Azithromycin | 1.4 - 250 | 2 | Egypt, Palestine |
| 12 | Bezafibrate | 195-480 | 3 | Jordan, Israel |
| 13 | Bisoprolol | 118 | 1 | Saudi Arabia |
| 14 | Carbamazepine | 41-17000 | 32 | Tunisia, Saudi Arabia, Jordan, Israel |
| 15 | Cephalexin | 7.5 - 1530 | 2 | Saudi Arabia, Tunisia |
| 16 | Chloramphenicol | 300-1100 | 2 | Tunisia |
| 17 | Ciprofloxacin | 51.2-987 | 5 | Egypt, Qatar, Palestine, Tunisia |
| 18 | Clarithromicyn | 22-875 | 2 | Saudi Arabia |
| 19 | Clavulanic acid | 14780-23650 | 2 | Qater |
| 20 | Clofibric acid | 10-150 | 3 | Jordan, Israel |
| 21 | Cotinine | 78 | 1 | Jordan |
| 22 | Diazepam | 404-720 | 2 | Jordan |
| 23 | Diclofenac | 70-40000 | 12 | Saudi Arabia, Jordan, Israel, Algeria |
| 24 | Dihydrostreptomycin | 400 | 1 | Tunisia |
| 25 | Dilantin | < 20-440 | 3 | Saudi Arabia |
| 26 | Diphenhydramine | 44-525 | 5 | Saudi Arabia, Jordan |
| 27 | Eprosartan | 80 | 1 | Saudi Arabia |
| 28 | Erythromycin | 2.4-1187 | 14 | Jordan, Qatar, Palestine, Tunisia |
| 29 | Fenofibrate | 260 | 1 | Jordan |
| 30 | Fenofibric acid | 160 | 2 | Jordan |
| 31 | Florfenicol | 100-800 | 4 | Tunisia |
| 32 | Fluoxetine | 13.5-295 | 4 | Saudi Arabia |
| 33 | Gemfibrosil | 7.5-4800 | 9 | Jordan, Saudi Arabia, Israel |
| 34 | Gentamycin c1 | 300-400 | 2 | Tunisia |
| 35 | Gentamycin c1a | 200-600 | 2 | Tunisia |
| 36 | Gentamycin c2 | 300 | 1 | Tunisia |
| 37 | Glimepiride | 2667-10667 | 2 | Jordan |
| 38 | Ibuprofen | < 10-40000 | 14 | Tunisia, Saudi Arabia, Jordan, Israel, Algeria |
| 39 | Kanamycin B | 700-5400 | 3 | Tunisia |
| 40 | Ketoprofen | 64-1034.5 | 3 | Algeria, Jordan, Israel |
| 41 | Labetalol | 3.44-19.05 | 2 | Emirate |
| 42 | Lamotrigine | 820 | 1 | Israel |
| 43 | Lidocaine | 114 | 1 | Saudi Arabia |
| 44 | Linezolid | 1 | 1 | Palestine |
| 45 | MDMA | <5 | 1 | Jordan |
| 46 | Metformin | 3190 | 1 | Saudi Arabia |
| 47 | Methotrexate | 124000-250667 | 3 | Jordan |
| 48 | Metoprolol | 10 | 1 | Israel |
| 49 | Metoprolol | 6.56-10.96 | 2 | Emirate |
| 50 | Metronidazole | 155-230 | 2 | Qatar |
| 51 | Morphine | <5-22 | 2 | Saudi Arabia, Jordan |
| 52 | NACS (Nacetylsulfamethoxazol) | 22 | 1 | Saudi Arabia |
| 53 | Naproxen | 2.94-1300 | 13 | Tunisia, Saudi Arabia, Jordan, Israel, Algeria |
| 54 | Neomycin | 400-11200 | 4 | Tunisia |
| 55 | Norfluoxetine | 7250 | 1 | Saudi Arabia |
| 56 | Ofloxacin | 190-648 | 7 | Tunisia |
| 57 | Oxacilin | 51 | 1 | Palestine |
| 58 | Oxolinic acid | 51.3 | 1 | Palestine |
| 59 | Oxprendol | 95 | 1 | Saudi Arabia |
| 60 | Paracetamol | 73-409 | 3 | Saudi Arabia, Tunisia |
| 61 | Paromycin | 900-1300 | 2 | Tunisia |
| 62 | Penicillin | 11-216 | 2 | Qatar |
| 63 | Penicillin G | 21.3 | 1 | Palestine |
| 64 | Phenazone | 11 | 1 | Jordan |
| 65 | Pipemidic acid | 23.9 | 1 | Palestine |
| 66 | Primidone | 7-645 | 4 | Saudi Arabia |
| 67 | Propranolol | 12.18-12.36 | 2 | Emirate |
| 68 | Sildenafil | 10 | 1 | Israel |
| 69 | Sisomycin | 3900-1000 | 3 | Tunisia |
| 70 | Streptomycin | 800-1200 | 2 | Tunisia |
| 71 | Sulfamethazine | 43 – 73.5 | 2 | Jordan, Tunisia |
| 72 | Sulfamethoxazole | 5.5 - 1720 | 17 | Egypt, Tunisia, Saudi Arabia, Jordan, Israel |
| 73 | Sulfapyridine | 20 | 1 | Israel |
| 74 | Spiramycin | 370.04 | 1 | Tunisia |
| 75 | Terbutaline | 7.53-11.11 | 2 | Emirate |
| 76 | Tetracycline | 92.5-210 | 3 | Qatar, Palestine |
| 77 | Thiabendazole | 17 | 1 | Jordan |
| 78 | Triclocarban | 27.3 | 1 | Palestine |
| 79 | Trimethoprim | <5-785 | 5 | Saudi Arabia, Jordan |
| 80 | Valsartan | 106-307 | 2 | Saudi Arabia |
| **Personal care products** |  |  |  |  |
| 1 | Methlyparabene | 40-443000 | 7 | Saudi Arabia, Tunisia |
| 2 | Oxybenzone | 28.5-380 | 4 | Saudi Arabia |
| 3 | Propylparaben | < 20-585 | 6 | Saudi Arabia, Tunisia |
| 4 | Triclocarban | 75-264.5 | 4 | Saudi Arabia |
| 5 | Triclosan | 100-74000 | 9 | Saudi Arabia, Palestine, Israel |
| **Hormones** |  |  |  |  |
| 1 | Estriol | 0.2-360 | 9 | Palestine, Tunisia, Israel |
| 2 | Estrone | 0.6-16.1 | 11 | Palestine, Israel |
| 3 | Testosterone | 1.2 | 1 | Israel |
| **Pesticides** |  |  |  |  |
| 1 | Acetamiprid | 25 | 1 | Saudi Arabia |
| 2 | Alachlor | 2.9 | 1 | Palestine |
| 3 | Atrazine | < 6-27 | 5 | Saudi Arabia, Palestine |
| 4 | Atrazine-deethyl | 81.2 | 1 | Saudi Arabia |
| 5 | Azoxystrobin | 25-600 | 2 | Saudi Arabia |
| 6 | Diazinon | 152-384 | 2 | Saudi Arabia |
| 7 | Heptachlor | 100 | 1 | Egypt |
| 8 | Imidacloprid | 25 | 1 | Saudi Arabia |
| 9 | Lindane | 190 | 1 | Egypt |
| 10 | Malathion | 10 | 1 | Saudi Arabia |
| 11 | p,p'-DDT | 470 | 1 | Egypt |
| 12 | Pirimicarb | 102 | 1 | Saudi Arabia |
| 13 | Terbuthylazine | 22-87 | 2 | Saudi Arabia |
| 14 | Trifluralin | 1 | 1 | Palestine |
| **Organic compounds** |  |  |  |  |
| 1 | 7 PCBs dioxin like | 280000 - 1180000 | 1 | Tunisia |
| 2 | 14 PAHs | 370000 - 830000 | 1 | Tunisia |
| **Plasticizers** |  |  |  |  |
| 1 | Atrazine | 7 | 1 | Saudi Arabia |
| 2 | Bis (2-ethylhexyl) phtalate | 340-935 | 5 | Saudi Arabia |
| 3 | Bisphenol A | < 20-6679 | 11 | Saudi Arabia, Israel |
| 4 | Bytyl benzyl phtalate | 108-660 | 5 | Saudi Arabia |
| 5 | Dibutylphtalate | 408-1037 | 5 | Saudi Arabia |
| 6 | Diethyl phtalate | 96-304 | 5 | Saudi Arabia |
| 7 | Dimethyl phtalate | 72-469 | 5 | Saudi Arabia |
| 8 | Dioctylphtalate | 15-420 | 5 | Saudi Arabia |
| **Phenols** |  |  |  |  |
| 1 | Anionic surfactant | 9600000-10600000 | 1 | Israel |
| 2 | nonionic detergents (alkyl phenol ethoxylate) | 23500-15000000 | 6 | Israel |
| **Illicit drugs** |  |  |  |  |
| 1 | Cocaine | 27-234 | 4 | Tunisia |

Table SI4: Concentration ranges, number of information points of each emerging pollutant detected in surface water in MENA region

| **Total number/group** | **Detected pollutants** | **Min- Max Concentration (ng/L)** | **Number of information points** | **Country** |
| --- | --- | --- | --- | --- |
| **Food additives** |  |  |  |  |
| 1 | Caffeine | 24-23000 | 14 | Lebanon, Jordan, Saudi Arabia, Tunisia |
| **Pharmaceuticals** |  |  |  |  |
| 1 | 1,7-Dimethylxanthine | 10-53 | 2 | Jordan |
| 2 | Acetaminophen | 36 | 1 | Jordan |
| 3 | Amoxicillin | 159-4107 | 1 | Morocco |
| 4 | Amphetamine | 18 | 1 | Jordan |
| 5 | Anhydro-erythromycine | 172 | 1 | Lebanon |
| 6 | Atenolol | 297 | 1 | Tunisia |
| 7 | Bezafibrate | 26-390 | 2 | Jordan |
| 8 | Carbamazepine | 200- 7500 | 6 | Jordan, Tunisia |
| 9 | Ciprofloxacin | 7.2 - 1058 | 1 | Morocco |
| 10 | Cotinine | 15 | 1 | Jordan |
| 11 | Clofibric acid | 31 (Cmax : 33) | 1 | Jordan |
| 12 | Danofloxacine | 85 | 1 | Lebanon |
| 13 | Diazinon | 1016 | 1 | Saudi Arabia |
| 14 | Diazepam | 13 (Cmax : 13) | 1 | Jordan |
| 15 | Diclofenac | 35-1390 | 5 | Algeria, Jordan, Saudi Arabia Tunisia |
| 16 | Erythromycin | 3-3240 | 4 | Morocco, Jordan, Lebanon |
| 17 | Erythromycine A enolether | 121 | 1 | Lebanon |
| 18 | Gemfibrozil | 150 - 1200) | 2 | Jordan |
| 19 | Ibuprofen | 80-1400 | 4 | Algeria, Jordan, Tunisia |
| 20 | Naproxen | 69-550 | 4 | Algeria, Jordan, Tunisia |
| 21 | Norﬂoxacin | 50 | 1 | Lebanon |
| 22 | Ofloxacine | 78 | 1 | Lebanon |
| 23 | Oxolinic acid | 12.2-64 | 1 | Morocco |
| 24 | Paracetamol | 3069 |  | Saudi Arabia |
| 25 | Phenazone | 50-59 | 2 | Jordan |
| 26 | Rifaximin | 155 | 1 | Lebanon |
| 27 | Sarafloxacine | 74 | 1 | Lebanon |
| 28 | Sulfamethazine | 8 to 65 | 3 | Jordan, Lebanon |
| 29 | Sulfamethoxazole | 1.9-553 | 5 | Jordan, Morocco, Tunisia |
| 30 | Sulfathiazole | 4100 | 1 | Lebanon |
| 31 | Tetracycline | 111.4-286 | 1 | Morocco |
| 32 | Trimethoprim | 1.9-264 | 1 | Morocco |
| **Personal care products** |  |  |  |  |
| 1 | Methylparabene | 1048 | 1 | Tunisia |
| 2 | Triclosan | 500 | 1 | Tunisia |
| 3 | Methylparaben | 54.6 | 1 | Egypt |
| 4 | Propylparaben | 21.3 | 1 | Egypt |
| 5 | Butylparaben | 7.2 | 1 | Egypt |
| **Pesticides** |  |  |  |  |
| 1 | Aametryne | 7.5 | 1 | Egypt |
| 2 | BHC | 390 | 1 | Egypt |
| 3 | Captan | 15.5-18.5 | 2 | Egypt |
| 4 | DDD | 1-1.29 | 2 | Lebanon |
| 5 | DDE | 1.1-23.25 | 4 | Lebanon |
| 6 | DDT | 0.1-1835 | 10 | Egypt |
| 7 | Dieldrin | 1 | 1 | Egypt |
| 8 | Dimethoate | 165-170 | 2 | Egypt |
| 9 | Endosulfan I | 198-453 | 2 | Egypt |
| 10 | Endosulfan II | 1 | 1 | Egypt |
| 11 | Endosulfan sulfate | 1-5.3 | 2 | Egypt, Lebanon |
| 12 | Endrin | 6.25 | 1 | Egypt |
| 13 | Endrin aldehyde | 1 | 1 | Egypt |
| 14 | HCB | < 0.001-217.5 | 10 | Egypt, Lebanon |
| 15 | HCH | 1388 | 1 | Egypt |
| 16 | Heptachlor | 65-700 | 3 | Egypt |
| 17 | Heptachlor epoxide | 180-380 | 2 | Egypt |
| 18 | Lindane | 7.8-1970 | 3 | Egypt, Lebanon |
| 19 | Malathion | 35.5-45.5 | 2 | Egypt |
| 20 | Methoxychlor | 1.8 | 1 | Lebanon |
| 21 | o,p' -DDT | 950 | 1 | Egypt |
| 22 | p,p'-DDT | 650-4110 | 2 | Egypt |
| 23 | Parathion-ethyl | 450 | 1 | Tunisia |
| 24 | Tetradifon | 6.6 | 1 | Lebanon |
| 25 | β -Endosulfan | 24.46-55.32 | 2 | Lebanon |
| 26 | γ-HCH | 0.06-332 | 8 | Egypt |
| 27 | Dichlorovores | 360600 | 1 | Iraq |
| 28 | Carbendazim (Benzimidazole) | 58 | 1 | Algeria |
| 29 | Propamocarb HCl (carbamate) | 47 | 1 | Algeria |
| 30 | Triazole | 0.03 - 24.27 | 1 | Tunisia |
| **Organic compounds** |  |  |  |  |
| 1 | 12 PAHs | 360 - 530 | 1 | Iraq |
| 2 | 13 PAHs | 7780000 – 53740000 | 1 | Iraq |
| 3 | 14 PAHs | 290-3273000 | 5 | Algeria, Jordan, Tunisia, Iraq |
| 4 | 16 PAHs | 2.25-1462 | 5 | Algeria, Oman, Iraq |
| 5 | 21 PAHs | 0.739 – 1.974 | 1 | Iraq |
| 6 | 24 PAHs | 403.4-725.8 | 2 | Tunisia |
| 7 | 26 PAHs | 4860-15000 | 2 | Tunisia |
| 8 | 7 PCBs dioxin like | 3-235000 | 3 | Algeria, Egypt, Tunisia |
| 9 | PCB52 | 63.52-64.75 | 2 | Egypt, Tunisia |
| 10 | PCBs Ar 1254 | 410-640.5 | 2 | Egypt |
| 11 | PCBs Ar 1242 | 39-321.5 | 2 | Egypt |
| 12 | PCBs Ar 1260 | 14-300 | 4 | Egypt |
| **Plasticizers** |  |  |  |  |
| 1 | Bisphenol A | 15.9-784 | 2 | Tunisia, Egypt |
| **Phenols** |  |  |  |  |
| 1 | 66 Phenols | 1820000-2090000 | 2 | Jordan |
| 2 | nonnionic surfactant | 130000-7850000 | 5 | Israel |
| **Microplastics** |  |  |  |  |
| **1** | Microplastics | 0.49 (items/m^3^) | 6 | Oman, Tunisia, Lebanon, Saudi Arabia |

Table SI5: Concentration ranges, number of information points of each emerging pollutant detected in groundwater in MENA region

| **Total number/group** | **Detected pollutants** | **Min- Max Concentration (ng/L)** | **Number of information points** | **Country** |
| --- | --- | --- | --- | --- |
| **Food additives** |  |  |  |  |
| 1 | Caffeine | 6-259 | 12 | Palestine, Lebanon |
| **Pharmaceuticals** |  |  |  |  |
| 1 | 1-OH-ibuprofene | 23-40 | 5 | Tunisia |
| 2 | Azithromycin | 4,7 | 1 | Palestine |
| 3 | Carbamazepine | 6.2 -1045 | 9 | Jordan, Israel, Tunisia |
| 4 | Cloﬁbric acid | 40-75 | 1 | Egypt |
| 5 | Ciprofloxacin | 23,8 | 1 | Palestine |
| 6 | Fenofibrate | 74-130 | 2 | Jordan |
| 7 | Ibuprofen | 56 - 65 | 2 | Jordan |
| 8 | Linezolid | 0,8 | 1 | Palestine |
| 9 | Oxolinic acid | 24 | 1 | Palestine |
| 10 | Penicillin G | 4,1 | 1 | Palestine |
| 11 | Pipemidic acid | 10,2 | 1 | Palestine |
| 12 | Sulfamethoxazole | 12.5-41.5 | 4 | Israel, Tunisia |
| **Personal care products** |  |  |  |  |
| 1 | Methlyparabene | 63-109 | 2 | Tunisia |
| 2 | Methlyparabene | 63 |  |  |
| 3 | Propylparabene | 30 | 1 | Tunisia |
| 4 | Triclosan | 22-289 | 5 | Tunisia |
| **Pesticides** |  |  |  |  |
| 1 | Alachlor | 3.3-204500 | 3 | Palestine, Israel, Algeria |
| 2 | Aldrin | 0.4-9330 | 3 | Lebanon |
| 3 | Atrazine | 12.1 | 2 | Palestine, Israel |
| 4 | Bendiocarb | 181000 | 1 | Saudi Arabia |
| 5 | Bromacil | 60750 | 1 | Israel |
| 6 | Chlorobenzilate | 1111 | 1 | Lebanon |
| 7 | Chloroneb | 3000-114000 | 3 | Saudi Arabia |
| 8 | Cypermethrin | 21000-37000 | 2 | Saudi Arabia |
| 9 | DDD | 1.6-2.8 | 2 | Lebanon |
| 10 | DDE | 1.1-30 | 3 | Lebanon |
| 11 | DDT | 22.5-340 | 3 | Egypt, Lebanon |
| 12 | Dieldrin | 0.6-9560 | 2 | Lebanon |
| 13 | Dimethoate | 5000-418000 | 13 | Saudi Arabia |
| 14 | Endosulfan sulfate | 18-791 | 2 | Lebanon, Morocco |
| 15 | Endrin | 8 - 3210.5 | 4 | Algeria, Egypt, Lebanon |
| 16 | Endrin Aldehyde | 83.05-1001 | 2 | Algeria, Lebanon |
| 17 | Endrin Ketone | 66-592 | 4 | Algeria, Egypt, Lebanon |
| 18 | HCB | 0.52-1.5 | 2 | Lebanon |
| 19 | HCH | févr-68 | 3 | Egypt, Lebanon |
| 20 | Heptachlor | 3375 | 1 | Lebanon |
| 21 | Hepachlor Epoxyde | 561,8 | 1 | Algeria |
| 22 | Heptachlor Epox.A + B | 740-9005 | 2 | Lebanon |
| 23 | Lindane | 12,9 | 1 | Algeria |
| 24 | Meolachlor | 36,7 | 1 | Algeria |
| 25 | Methomyl | 4000-97000 | 8 | Saudi Arabia |
| 26 | Methoxychlor | 0.08-57 | 2 | Saudi Arabia |
| 27 | p,p-DDE | 7500-90000 | 2 | Saudi Arabia |
| 28 | p,p-DDT | 107000 | 1 | Saudi Arabia |
| 29 | Prometryn | 37750 | 1 | Israel |
| 30 | Promoxenyl | 74000 | 1 | Saudi Arabia |
| 31 | Tetradifon | 0.2 | 1 | Lebanon |
| 32 | Toxaphen | 2200-8000 | 2 | Saudi Arabia |
| 33 | Triclocarban | 47.2 | 1 | Palestine |
| 34 | Trifluralin | 2 | 1 | Palstine |
| 35 | α-HCH | 1460-1787 | 2 | Lebanon |
| 36 | β-Endosulfan | 5-280 | 2 | Lebanon, Morocco |
| 37 | β-HCH | 126-160 | 2 | Lebanon |
| 38 | γ-HCH | 311-1304 | 2 | Lebanon |
| 39 | δ-HCH | 17-423 | 2 | Lebanon |
| Organic compounds |  |  |  |  |
| 1 | 1,2,3-Trichlorobenzene | 30000-3390000 | 3 | Saudi Arabia |
| 2 | 1,2-Dibromoethane | 100000-780000 | 2 | Saudi Arabia |
| 3 | 1,3-Dichlorobenzene | 50000-990000 | 2 | Saudi Arabia |
| 4 | 1,4-Dichlorobenzene | 90000-2050000 | 1 | Saudi Arabia |
| 5 | 14 PAH | 20400 - 1930000 | 1 | Tunisia |
| 6 | 1-Chlorobutane | 70000 | 1 | Saudi Arabia |
| 7 | 2,2-Dichloropropane | 70000 | 1 | Saudi Arabia |
| 8 | 2-Chlorotoluene | 70000-3700000 | 1 | Saudi Arabia |
| 9 | 7 PCBs | 5200 - 196000 | 1 | Saudi Arabia |
| 10 | Allylchloride | 660000-1500000 | 1 | Saudi Arabia |
| 11 | Benzene | 280000-6900000 | 4 | Saudi Arabia |
| 12 | Bromobenzene(bromoform) | 10000-520000 | 1 | Saudi Arabia |
| 13 | Bromochloromethane | 40000-2520000 | 2 | Saudi Arabia |
| 14 | Bromodichloromethane | 1280000-2500000 | 1 | Saudi Arabia |
| 15 | Butylbenzene | 680000 | 1 | Saudi Arabia |
| 16 | Chlorobenzene | 120000-2770000 | 2 | Saudi Arabia |
| 17 | Ethylbenzene | 780000-800000 | 2 | Saudi Arabia |
| 18 | Ethylmethacrylate | 940000 | 1 | Saudi Arabia |
| 19 | Hexachlorpentadiene | 2000 | 1 | Saudi Arabia |
| 20 | Indeno(1,2,3)pyrene | 10000–110000 | 1 | Saudi Arabia |
| 21 | Isopropylbenzene | 150000-710000 | 1 | Saudi Arabia |
| 22 | Methylenechloride | 40000-260000 | 1 | Saudi Arabia |
| 23 | Methylmethacrylate | 360000 | 1 | Saudi Arabia |
| 24 | Methyltetra-butylether(MTBE) | 210000 | 1 | Saudi Arabia |
| 25 | m-Xylene | 320000 | 1 | Saudi Arabia |
| 26 | n-Butylbenzene | 60000-1680000 | 2 | Saudi Arabia |
| 27 | o-Xylene | 20000-360000 | 1 | Saudi Arabia |
| 28 | Propionitrile | 1850000 | 1 | Saudi Arabia |
| 29 | P-Xylene | 1320000-1880000 | 1 | Saudi Arabia |
| **Plasticizers** |  |  |  |  |
| 1 | Bisphenol A | 6.4-189 | 3 | Tunisia, Egypt |
| **Phenols** |  |  |  |  |
| 1 | 2,4-Dinitrophenol | 235000 | 1 | Saudi Arabia |
| 2 | 2,4,6-Tricholoro-phenol | 833000 | 1 | Saudi Arabia |
| 3 | 2-Methyle-4,6-dinitrophenol | 936500 | 1 | Saudi Arabia |
| 4 | 2,4-Dimethylphenol | 24000 | 1 | Saudi Arabia |
| 5 | 2-Nitrophenol | 22000 | 1 | Saudi Arabia |
| 6 | 4-Nitrophenol | 550000 | 1 | Saudi Arabia |
| 7 | alkylphenolethoxylates | 390000 | 1 | Saudi Arabia |
| 8 | Toluene | 200000-1620000 | 3 | Saudi Arabia, Israel |
| 9 | C2-Benzene | 700000 | 1 | Saudi Arabia |
| 10 | C3-Benzene | 60000 | 1 | Saudi Arabia |
| 11 | Phtalates | 300000 | 1 | Israel |

Table SI6: Concentration ranges, number of information points of each emerging pollutant detected in drinking water in MENA region

| **Total number/group** | **Detected pollutants** | **Min- Max Concentration (ng/L)** | **Number of information points** | **Country** |
| --- | --- | --- | --- | --- |
| **Pharmaceuticals** |  |  |  |  |
| 1 | Ibuprofen | 312 | 1 | Algeria |
| 2 | ketoprofen | 273 | 1 | Algeria |
| 3 | Ciprofloxacin | 1312 (finished water)  1270-1344 (raw water) | 1 | Iraq |
| 4 | Levofloxacin | 177 – 414 (raw water) | 1 | Iraq |
| 5 | Amoxicillin | 1500 (raw water) | 1 | Iraq |
| **Organic compounds** |  |  |  |  |
| 1 | 16 PAHs | 8.46 - 3654.8 | 1 | Iraq |
| 2 | 16 PAHs | 80 - 1462 | 1 | Iraq |
| **Plasticizers** |  |  |  |  |
| 1 | BPA | 2230 (Cmax)  36.1 (Cavg) | 1 | Egypt |
| 2 | BPA | 290–41,190 | 1 | Saudi Arabia |
| **PCPs** |  |  |  |  |
| 1 | Methylparaben | 1160 (Cmax)  47.2 (Cavg) | 1 | Egypt |
| 2 | Propylparaben | 590 (Cmax) | 1 | Egypt |
| 3 | Butylparaben | 6380 (Cmax)  25.8 (Cavg) | 1 | Egypt |

Table SI7: The percent removals of emerging pollutants described in the bibliographic database for MENA region

| **Country** | **Location** | **Pollutants** | **Removal efficiency: Secondary treatment (%)** | **Removal efficiency: Tertiary treatment (%)** | **Treatment process** | **Reference** |
| --- | --- | --- | --- | --- | --- | --- |
| Jordan | AS-Samra WWTP | 1,7-Dimethylxanthine | 99.9 | - | Activated sludge/extended aeration since 2015 | Al-Mashaqbeh et al., 2019 |
| Israel | Yad Hana | 17β-estradiol | 100 | - | Aeration pond | Dotan et al,, 2016 |
| Israel | Shafdan | 17β-estradiol | 100 | - | **Secondary treatment**: Activated sludge/bottom aeration  **Additional treatment:** Soil-aquifer treatment | Dotan et al,, 2016 |
| Saudi arabia | AlmadinahAlmunawarah | Acetaminophen | 19.8 | - | **Secondary treatment**:  N/A  **Tertiary treatment**: chlorination | Shraim et al., 2012 |
| Jordan | AS-Samra WWTP | Acetaminophen | 99.9 | - | Activated sludge/extended aeration system since 2015 | Al-Mashaqbeh et al., 2019 |
| Tunisia | WWTPs Tunis-nord | Amikacin | 14 | - | Activated sludge | Tahrani et al., 2016 |
| Tunisia | WWTPs Ben Arous | Amikacin | 57 | - | Activated sludge | Tahrani et al., 2016 |
| Qatar | WWTP, Qatar | Amoxicillin |  | 46.3 | Old WWTP  **Secondary treatment**:  Conventional aeration and activated sludge recirculation  **Tertiary treatment**:  Sand ﬁlter and Disinfection with Chlorine | Al-Maadheed et al,, 2019 |
| Qatar | WWTP, Qatar | Amoxicillin |  | 45.1 | New WWTP  **Secondary treatment**:  Anaerobic Selector for phosphorous removal, Anoxic Selector for Nitrogen Removal and extended aeration with activated sludge recirculation **Tertiary treatment**:  Sand ﬁlter and ultraﬁltration and Disinfection with Chlorine | Al-Maadheed et al,, 2019 |
| Jordan | AS-Samra WWTP | Amphetamine | 80.2 | - | activated sludge/extended aeration system since 2015 | Al-Mashaqbeh et al., 2019 |
| Tunisia | WWTPs Tunis-nord | Aparamycin | 67 | - | Activated sludge | Tahrani et al., 2016 |
| Tunisia | WWTPs Charguia | Aparamycin | 67 | - | Activated sludge | Tahrani et al., 2016 |
| Tunisia | WWTPs Ben Arous | Aparamycin | 100 | - | Activated sludge | Tahrani et al., 2016 |
| Saudi arabia | Two HWWTPs, Riyadh | Atenolol |  | 89 | Hospital WWTPs  **Secondary treatment:**  Activated sludge  **Tertiary treatment**:  Sandfiltrationanddisinfectionwith Chlorine | Al Qarni et al., 2016 |
| Saudi arabia | AlmadinahAlmunawarah | Atenolol |  | 73 | **Secondary treatment**:  N/A  **Tertiary treatment**: Disinfection with Chlorine | Shraim et al., 2012 |
| Tunisia | Seven Tunisian WWTPs | Atenolol | 20 | - | Activated sludge | Moslah et al., 2017 |
| Palestine | Nablus and Jericho Governorate | Azithromycin | 97.50 | - | Small-scale, off-grid greywater treatment systems | Craddock et al., 2020 |
| Palestine | El Beireh | Bisphenol A | 100 | - | Secondary treatment: Activated sludge/Bottom aeration. | Dotan et al,, 2016 |
| Israel | Yad Hana | Bisphenol A | 100 | - | Secondary treatment: Aeration pond. | Dotan et al,, 2016 |
| Israel | Ra'anana | Bisphenol A | 100 | - | **Secondary**treatment: Sequence bath reactor  **Tertiarytreatment:** Sand filtration | Dotan et al,, 2016 |
| Israel | Ben-Gurion airport | Bisphenol A | 100 | - | Tertiary treatment: membrane bioreactor. | Dotan et al,, 2016 |
| Israel | Shafdan | Bisphenol A | 100 | - | **Secondary treatment**: Activated sludge/bottom aeration  **Additional treatment:** Soil-aquifer treatment | Dotan et al,, 2016 |
| Israel | HodHasharon | Bisphenol A | 100 | - | **Secondary treatment**:  Activated sludge/bottom aeration  **Tertiary treatment:**  Sand filtration + Disinfection with UV. | Dotan et al,, 2016 |
| Israel | Yeruham | Bisphenol A | 80.95 | - | **Secondary treatment**:  Activated sludge/Rotating disks  **Tertiary treatment**: sand filtration. | Dotan et al,, 2016 |
| Saudi arabia | Two HWWTPs, Riyadh | caffeine |  | >99 | Hospital WWTPs  **Secondary treatment:**  Activated sludge  **Tertiary treatment**:  Sandfiltrationanddisinfection with chlorine | Al Qarni et al., 2016 |
| Palestine | Nablus and Jericho Governorate | Caffeine | 69 | - | Small-scale, off-grid greywater treatment systems | Craddock et al., 2020 |
| Tunisia | Seven Tunisian WWTPs | Caffeine | 65 | - | Activated sludge | Moslah et al., 2017 |
| Jordan | Abu-Nusair WWTPs | Caffeine | 65 | - | Rotating biological contractors with extended aeration | Alahmad et al., 2010 |
| Jordan | WadiAlseir WWTPs | Caffeine | 25 | - | Rotating biological contractors with extended aeration | Alahmad et al., 2010 |
| Jordan | Baqa'a WWTPs | Caffeine | 62 | - | Rotating biological contractors with extended aeration | Alahmad et al., 2010 |
| Jordan | AS-Samra WWTP | Caﬀeine | 99.9 | - | activated sludge/extended aeration system since 2015 | Al-Mashaqbeh et al., 2019 |
| Saudi arabia | Two HWWTPs, Riyadh | carbamazepine |  | >86 | Hospital WWTPs  **Secondary treatment:**  Activated sludge  **Tertiary treatment**:  Sandfiltrationanddisinfection with chlorine | Al Qarni et al., 2016 |
| Tunisia | SixTunisian WWTPs | carbamazepine | -40 | - | Activated sludge | Moslah et al., 2017 |
| Tunisia | Tunisian WWTPs, Lagoon of Korba | carbamazepine |  | -78 | **Secondary treatment:**  Activated sludge  **Tertiary treatment**:  Aerated lagoon | Moslah et al., 2017 |
| Jordan | Abu-Nsair WWTP, Jordan | carbamazepine | 27 | - | Rotating biological contractors with extended aeration | Al-Tarawneh et al., 2014 |
| Jordan | Al-Aqaba WWTP, Jordan | carbamazepine |  | 33 | **Secondary treatment:**  Activated sludge  **Extensive**  **systems**:  waste stabilization pond | Al-Tarawneh et al., 2014 |
| Jordan | Al-Karak WWTP | carbamazepine | 75 | - | Trickling filter | Al-Tarawneh et al., 2014 |
| Jordan | Al-Salt WWTP, Jordan | carbamazepine | -43 | - | Rotating biological contractors with extended aeration | Al-Tarawneh et al., 2014 |
| Jordan | Irbid WWTP, Jordan | carbamazepine | 60 | - | Trickling filter | Al-Tarawneh et al., 2014 |
| Jordan | Kherbet As-Samra WWTP, Jordan | carbamazepine |  | 0 | **Secondary treatment:**  Activated sludge  **Tertiary treatment**:  waste stabilization pond | Al-Tarawneh et al., 2014 |
| Jordan | AS-Samra WWTP | Carbamazepine | 22.5 | - | Activated sludge/extended aeration system since 2015 | Al-Mashaqbeh et al., 2019 |
| Saudi arabia | AlmadinahAlmunawarah | Cephalexin | 18.6 | - | **Secondary treatment**:  N/A  **Tertiary treatment**: Disinfection with Chlorine | Shraim et al., 2012 |
| Tunisia | WWTPs Tunis-nord | Chloramphenicol | 67 | - | Activated sludge | Tahrani et al., 2016 |
| Tunisia | WWTPs Chotrana | Chloramphenicol | 40 | - | Activated sludge | Tahrani et al., 2016 |
| Qatar | WWTP, Qatar | Ciprofloxacin |  | 70.5 | Old WWTP  **Secondary treatment**:  Conventional aeration and activated sludge recirculation  **Tertiary treatment**:  Sand ﬁlter and Disinfection with Chlorine | Al-Maadheed et al,, 2019 |
| Qatar | WWTP, Qatar | Ciprofloxacin |  | 26 | New WWTP  **Secondary treatment**:  Anaerobic Selector for phosphorous removal, Anoxic Selector for Nitrogen Removal and extended aeration with activated sludge recirculation **Tertiary treatment**:  Sand ﬁlter and ultraﬁltration and Disinfection with Chlorine | Al-Maadheed et al,, 2019 |
| Saudi arabia | HWWTP 1 and 2, Riyadh | ciprofloxacin |  | >99 | Hospital WWTPs  **Secondary treatment:**  Activated sludge  **Tertiary treatment**:  Sandfiltrationanddisinfection with Chlorine | Al Qarni et al., 2016 |
| Palestine | Nablus and Jericho Governorate | Ciprofloxacin | -7 | - | Small-scale, off-grid greywater treatment systems | Craddock et al., 2020 |
| Tunisia | Seven Tunisian WWTPs | Ciprofloxacin | 100 | - | Activated sludge | Moslah et al., 2017 |
| Saudi arabia | Two HWWTPs, Riyadh | clarithromycin |  | 86 | Hospital WWTPs  **Secondary treatment:**  Activated sludge  **Tertiary treatment**:  Sandfiltrationanddisinfectionwith Chlorine | Al Qarni et al., 2016 |
| Tunisia | Seven Tunisian WWTPs | clarithromycin | 100 | - | Activated sludge | Moslah et al., 2017 |
| Jordan | AS-Samra WWTP | Cotinine | 98.4 | - | Activated sludge/extended aeration system since 2015 | Al-Mashaqbeh et al., 2019 |
| Jordan | Abu-Nusair WWTPs | Diclofenac | 100 | - | Rotating biological contractors with extended aeration | Alahmad et al., 2010 |
| Jordan | WadiAlseir WWTPs | Diclofenac | 44 | - | Rotating biological contractors with extended aeration | Alahmad et al., 2010 |
| Jordan | Baqa'a WWTPs | Diclofenac | 100 | - | Rotating biological contractors with extended aeration | Alahmad et al., 2010 |
| Jordan | Abu-Nsair WWTP, Jordan | diclofenac | 100 | - | Rotating biological contractors with extended aeration | Al-Tarawneh et al., 2014 |
| Jordan | Al-Aqaba WWTP, Jordan | diclofenac |  | 100 | **Secondary treatment:**  Activated sludge  **Tertiary treatment**:  waste stabilization pond | Al-Tarawneh et al., 2014 |
| Jordan | Al-Karak WWTP | diclofenac | 66 | - | Trickling filter | Al-Tarawneh et al., 2014 |
| Jordan | Al-Salt WWTP, Jordan | diclofenac | 93 | - | Rotating biological contractors with extended aeration | Al-Tarawneh et al., 2014 |
| Jordan | Irbid WWTP, Jordan | diclofenac | 75 | - | Trickling filter | Al-Tarawneh et al., 2014 |
| Jordan | Kherbet As-Samra WWTP, Jordan | diclofenac |  | 65 | **Secondary treatment:**  Activated sludge  **Tertiary treatment**:  waste stabilization pond | Al-Tarawneh et al., 2014 |
| Algeria | Beni Messous WWTP | Diclofenac | -173.7 | - | Activated sludge | Kermia et al., 2016 |
| Algeria | Reghaia WWTP | Diclofenac | 30.3 | - | Activated sludge | Kermia et al., 2016 |
| Tunisia | WWTPs Tunis-nord | Dihydrostreptomycin | 56 | - | Activated sludge | Tahrani et al., 2016 |
| Jordan | AS-Samra WWTP | Diphenhydramine | -770.5 | - | activated sludge/extended aeration system since 2015 | Al-Mashaqbeh et al., 2019 |
| Qatar | WWTP, Qatar | Erythromycin |  | 90.9 | Old WWTP  **Secondary treatment**:  Conventional aeration and activated sludge recirculation  **Tertiary treatment**:  Sand ﬁlter and Disinfection with Chlorine | Al-Maadheed et al,, 2019 |
| Qatar | WWTP, Qatar | Erythromycin |  | 86.1 | New WWTP  **Secondary treatment**:  Anaerobic Selector for phosphorous removal, Anoxic Selector for Nitrogen Removal and extended aeration with activated sludge recirculation **Tertiary treatment**:  Sand ﬁlter and ultraﬁltration and Disinfection with Chlorine | Al-Maadheed et al,, 2019 |
| Palestine | Nablus and Jericho Governorate | Erythromycin | -60 | - | Small-scale, off-grid greywater treatment systems | Craddock et al., 2020 |
| Tunisia | Seven Tunisian WWTPs | Erythromycin | -25 | - | Activated sludge | Moslah et al., 2017 |
| Jordan | Abu-Nsair WWTP, Jordan | erythromycin | 100 | - | Rotating biological contractors with extended aeration | Al-Tarawneh et al., 2014 |
| Jordan | Al-Aqaba WWTP, Jordan | erythromycin |  | 100 | **Secondary treatment:**  Activated sludge  **Tertiary treatment**:  waste stabilization pond | Al-Tarawneh et al., 2014 |
| Jordan | Al-Karak WWTP | erythromycin | 60 | - | Trickling filter | Al-Tarawneh et al., 2014 |
| Jordan | Al-Salt WWTP, Jordan | erythromycin | 20 | - | Rotating biological contractors with extended aeration | Al-Tarawneh et al., 2014 |
| Jordan | Irbid WWTP, Jordan | erythromycin | 75 | - | Trickling filter | Al-Tarawneh et al., 2014 |
| Jordan | Kherbet As-Samra WWTP, Jordan | erythromycin |  | 17 | **Secondary treatment:**  Activated sludge  **Tertiary treatment**:  waste stabilization pond | Al-Tarawneh et al., 2014 |
| Palestine | El Beireh | Estriol | 98.4 | - | Secondary treatment: Activated sludge- Bottom aeration. | Dotan et al,, 2016 |
| Palestine | Nablus | Estriol | 100 | - | Secondary treatment: Activated sludge- Bottom aeration. | Dotan et al,, 2016 |
| Israel | Yad Hana | Estriol | 97.6 |  | Secondary treatment: Aeration pond. | Dotan et al,, 2016 |
| Israel | Ra'anana | Estriol | 98.9 | 100 | **Secondary** treatment: Sequence bath reactor  **Tertiary** treatment: Sand filtration | Dotan et al,, 2016 |
| Israel | Ben-Gurion airport | Estriol |  | 100 | Tertiary treatment: membrane bioreactor. | Dotan et al,, 2016 |
| Israel | Shafdan | Estriol | 87.2 | 100 | **Secondary treatment**: Activated sludge/bottom aeration  **Additional treatment:** Soil-aquifer treatment | Dotan et al,, 2016 |
| Israel | HodHasharon | Estriol | 93.9 | 98 | **Secondary treatment**:  Activated sludge/bottom aeration  **Tertiary treatment:**  Sand filtration + Disinfection with UV. | Dotan et al,, 2016 |
| Israel | Yeruham | Estriol | 100 | - | **Secondary treatment**:  Activated sludge/Rotating disks  **Tertiary treatment**: sand filtration. | Dotan et al,, 2016 |
| Palestine | El Beireh | Estrone | 97 | - | Secondary treatment: Activated sludge- Bottom aeration. Water reuse: | Dotan et al,, 2016 |
| Palestine | Nablus | Estrone | 96.7 | - | Secondary treatment: Activated sludge- Bottom aeration. | Dotan et al,, 2016 |
| Israel | Yad Hana | Estrone | 93.2 | - | Secondary treatment: Aeration pond. | Dotan et al,, 2016 |
| Israel | Ra'anana | Estrone | 93.5 | 100 | **Secondary** treatment: Sequence bath reactor  **Tertiary** treatment: Sand filtration | Dotan et al,, 2016 |
| Israel | Ben-Gurion airport | Estrone | 98.8 | 98 | **Secondarytreatment:**  Activated Sludge- bottom aeration  **Tertiary treatment:** membrane bioreactor. | Dotan et al,, 2016 |
| Israel | Shafdan | estrone | 74.6 | 100 | **Secondary treatment**: Activated sludge/bottom aeration  **Additional treatment:** Soil-aquifer treatment | Dotan et al,, 2016 |
| Israel | HodHasharon | estrone | 90.9 | 94.7 | **Secondary treatment**:  Activated sludge/bottom aeration  **Tertiary treatment:**  Sand filtration + Disinfection with UV. | Dotan et al,, 2016 |
| Israel | Yeruham | estrone | 95.5 | 97.1 | **Secondary treatment**:  Activated sludge/Rotating disks  **Tertiary treatment**: sand filtration. | Dotan et al,, 2016 |
| Tunisia | WWTPs Tunis-nord | Florfenicol | 76 | - | Activated sludge | Tahrani et al., 2016 |
| Tunisia | WWTPs Charguia | Florfenicol | 33 | - | Activated sludge | Tahrani et al., 2016 |
| Tunisia | WWTPs Chotrana | Florfenicol | 57 | - | Activated sludge | Tahrani et al., 2016 |
| Tunisia | WWTPs Ben Arous | Florfenicol | 89 | - | Activated sludge | Tahrani et al., 2016 |
| Tunisia | WWTPs Tunis-nord | Gentamycin c1 | 63 | - | Activated sludge | Tahrani et al., 2016 |
| Tunisia | WWTPs Charguia | Gentamycin c1 | 50 | - | Activated sludge | Tahrani et al., 2016 |
| Tunisia | WWTPs Ben Arous | Gentamycin c1 | 100 | - | Activated sludge | Tahrani et al., 2016 |
| Tunisia | WWTPs Tunis-nord | Gentamycin c1a | 63 | - | Activated sludge | Tahrani et al., 2016 |
| Tunisia | WWTPs Charguia | Gentamycin c1a | 60 | - | Activated sludge | Tahrani et al., 2016 |
| Tunisia | WWTPs Tunis-nord | Gentamycin c2 | 70 | - | Activated sludge | Tahrani et al., 2016 |
| Qatar | WWTP, Qatar | Glavulanic acid |  | 65.7 | Old WWTP  **Secondary treatment**:  Conventional aeration and activated sludge recirculation  **Tertiary treatment**:  Sand ﬁlter and Disinfection with Chlorine | Al-Maadheed et al,, 2019 |
| Qatar | WWTP, Qatar | Glavulanic acid |  | 40.4 | New WWTP  **Secondary treatment**:  Anaerobic Selector for phosphorous removal, Anoxic Selector for Nitrogen Removal and extended aeration with activated sludge recirculation **Tertiary treatment**:  Sand ﬁlter and ultraﬁltration and Disinfection with Chlorine | Al-Maadheed et al,, 2019 |
| Jordan | Abu-Nusair WWTPs | Glimepiride | 72 | - | Rotating biological contractors with extended aeration | Alahmad et al., 2010 |
| Jordan | WadiAlseir WWTPs | Glimepiride | 81 | - | Rotating biological contractors with extended aeration | Alahmad et al., 2010 |
| Jordan | Baqa'a WWTPs | Glimepiride | 100 | - | Rotating biological contractors with extended aeration | Alahmad et al., 2010 |
| Jordan | Abu-Nusair WWTPs | Ibuprofen | 100 | - | Rotating biological contractors with extended aeration | Alahmad et al., 2010 |
| Jordan | WadiAlseir WWTPs | Ibuprofen | 38 | - | Rotating biological contractors with extended aeration | Alahmad et al., 2010 |
| Jordan | Baqa'a WWTPs | Ibuprofen | 75 | - | Rotating biological contractors with extended aeration | Alahmad et al., 2010 |
| Jordan | Al-Aqaba WWTP, Jordan | ibuprofen |  | 100 | **Secondary treatment:**  Activated sludge  **Tertiary treatment**:  waste stabilization pond | Al-Tarawneh et al., 2014 |
| Jordan | Al-Karak WWTP | ibuprofen | 61 | - | Trickling filter | Al-Tarawneh et al., 2014 |
| Jordan | Irbid WWTP, Jordan | ibuprofen | 43 | - | Trickling filter | Al-Tarawneh et al., 2014 |
| Jordan | Kherbet As-Samra WWTP, Jordan | ibuprofen |  | 100 | **Secondary treatment:**  Activated sludge  **Tertiary treatment**:  waste stabilization pond | Al-Tarawneh et al., 2014 |
| Algeria | Beni Messous WWTP | Ibuprofen | 95 | - | Activated sludge | Kermia et al., 2016 |
| Algeria | Reghaia WWTP | Ibuprofen | 78.8 | - | Activated sludge | Kermia et al., 2016 |
| Tunisia | WWTPs Tunis-nord | Kanamycin B | 28 | - | Activated sludge | Tahrani et al., 2016 |
|  |  |  |  |  |  |  |
| Tunisia | WWTPs Charguia | Kanamycin B | 42 | - | Activated sludge | Tahrani et al., 2016 |
| Tunisia | WWTPs Chotrana | Kanamycin B | 30 | - | Activated sludge | Tahrani et al., 2016 |
| Tunisia | WWTPs Ben Arous | Kanamycin B | 100 | - | Activated sludge | Tahrani et al., 2016 |
| Algeria | Beni Messous WWTP | Ketoprofen | -83 | - | Activated sludge | Kermia et al., 2016 |
| Saudi arabia | Two HWWTPs, Riyadh | Lidocaine |  | 64 | Hospital WWTPs  **Secondary treatment:**  Activated sludge  **Tertiary treatment**:  Sandfiltrationanddisinfection with chlorine | Al Qarni et al., 2016 |
| Palestine | Nablus and Jericho Governorate | Linezolid | 41 | - | Small-scale, off-grid greywater treatment systems | Craddock et al., 2020 |
| Jordan | AS-Samra WWTP | MDMA | 56.5 | - | activated sludge/extended aeration system since 2015 | Al-Mashaqbeh et al., 2019 |
| Saudi arabia | AlmadinahAlmunawarah | Metformin | >99 | - | **Secondary treatment**:  N/A  **Tertiary treatment**: disinfection with chlorine | Shraim et al., 2012 |
| Jordan | Abu-Nusair WWTPs | Methotrexate | 25 | - | Rotating biological contractors with extended aeration | Alahmad et al., 2010 |
| Jordan | WadiAlseir WWTPs | Methotrexate | 56 | - | Rotating biological contractors with extended aeration | Alahmad et al., 2010 |
| Jordan | Baqa'a WWTPs | Methotrexate | 27 | - | Rotating biological contractors with extended aeration | Alahmad et al., 2010 |
| Qatar | WWTP, Qatar | Metronidazole |  | 75.4 | Old WWTP  **Secondary treatment**:  Conventional aeration and activated sludge recirculation  **Tertiary treatment**:  Sand ﬁlter and Disinfection with Chlorine | Al-Maadheed et al,, 2019 |
| Qatar | WWTP, Qatar | Metronidazole |  | 86.6 | New WWTP  **Secondary treatment**:  Anaerobic Selector for phosphorous removal, Anoxic Selector for Nitrogen Removal and extended aeration with activated sludge recirculation **Tertiary treatment**:  Sand ﬁlter and ultraﬁltration and Disinfection with Chlorine | Al-Maadheed et al,, 2019 |
| Jordan | AS-Samra WWTP | Morphine | 89 | - | activated sludge/extended aeration system since 2015 | Al-Mashaqbeh et al., 2019 |
| Saudi arabia | Two HWWTPs, Riyadh | NACS |  | 97 | Hospital WWTPs  **Secondary treatment:**  Activated sludge  **Tertiary treatment**:  Sandfiltrationanddisinfectionwith Chlorine | Al Qarni et al., 2016 |
| Jordan | Abu-Nsair WWTP, Jordan | naproxen | 100 | - | Rotating biological contractors with extended aeration | Al-Tarawneh et al., 2014 |
| Jordan | Al-Aqaba WWTP, Jordan | naproxen |  | 100 | **Secondary treatment:**  Activated sludge  **Tertiary treatment**:  waste stabilization pond | Al-Tarawneh et al., 2014 |
| Jordan | Al-Karak WWTP | naproxen | 72 | - | Trickling filter | Al-Tarawneh et al., 2014 |
| Jordan | Al-Salt WWTP, Jordan | naproxen | 100 | - | Rotating biological contractors with extended aeration | Al-Tarawneh et al., 2014 |
| Jordan | Irbid WWTP, Jordan | naproxen | 57 | - | Trickling filter | Al-Tarawneh et al., 2014 |
| Jordan | Kherbet As-Samra WWTP, Jordan | naproxen |  | 100 | **Secondary treatment:**  Activated sludge  **Tertiary treatment**:  waste stabilization pond | Al-Tarawneh et al., 2014 |
| Algeria | Beni Messous WWTP | Naproxen | 72.6 | - | Activated sludge | Kermia et al., 2016 |
| Tunisia | WWTPs Tunis-nord | Neomycin | 62 | - | Activated sludge | Tahrani et al., 2016 |
| Tunisia | WWTPs Charguia | Neomycin | 32 | - | Activated sludge | Tahrani et al., 2016 |
| Tunisia | WWTPs Chotrana | Neomycin | 66 | - | Activated sludge | Tahrani et al., 2016 |
| Tunisia | WWTPs Ben Arous | Neomycin | 78 | - | Activated sludge | Tahrani et al., 2016 |
| Saudi arabia | AlmadinahAlmunawarah | Norfluoxetine | -3 | - | **Secondary treatment**:  N/A  **Tertiary treatment**: Disinfection with Chlorine | Shraim et al., 2012 |
| Israel | Yad Hana | Octylphenol | 100 | - | Secondary treatment: Aeration pond. | Dotan et al,, 2016 |
| Israel | Ra'anana | Octylphenol | 100 | - | **Secondary** treatment: Sequence bath reactor  **Tertiary** treatment: Sand filtration | Dotan et al,, 2016 |
| Israel | Ben-Gurion airport | Octylphenol | 100 | - | Tertiary treatment: membrane bioreactor. | Dotan et al,, 2016 |
| Israel | Shafdan | Octylphenol | 100 | - | **Secondary treatment**: Activated sludge/bottom aeration  **Additional treatment:** Soil-aquifer treatment | Dotan et al,, 2016 |
| Israel | HodHasharon | Octylphenol | 100 | - | **Secondary treatment**:  Activated sludge/bottom aeration  **Tertiary treatment:**  Sand filtration + Disinfection withUv. | Dotan et al,, 2016 |
| Tunisia | Seven Tunisian WWTPs | Ofloxacin | 7 | - | Activated sludge | Moslah et al., 2017 |
| Palestine | Nablus and Jericho Governorate | Oxacilin | 14.80 | - | Small-scale, off-grid greywater treatment systems | Craddock et al., 2020 |
| Palestine | Nablus and Jericho Governorate | Oxolinic acid | -43 | - | Small-scale, off-grid greywater treatment systems | Craddock et al., 2020 |
| Saudi arabia | Two HWWTPs, Riyadh | Paracetamol |  | >98 | Hospital WWTPs  **Secondary treatment:**  Activated sludge  **Tertiary treatment**:  Sandfiltrationanddisinfectionwith Chlorine | Al Qarni et al., 2016 |
| Tunisia | Seven Tunisian WWTPs | Paracetamol | 65 | - | Activated sludge | Moslah et al., 2017 |
| Tunisia | WWTPs Tunis-nord | Paromycin | 69 | - | Activated sludge | Tahrani et al., 2016 |
| Tunisia | WWTPs Ben Arous | Paromycin | 25 | - | Activated sludge | Tahrani et al., 2016 |
| Qatar | WWTP, Qatar | Penicillin |  | 95.6 | Old WWTP  **Secondary treatment**:  Conventional aeration and activated sludge recirculation  **Tertiary treatment**:  Sand ﬁlter and Disinfection with Chlorine | Al-Maadheed et al,, 2019 |
| Qatar | WWTP, Qatar | Penicillin |  | 0 | New WWTP  **Secondary treatment**:  Anaerobic Selector for phosphorous removal, Anoxic Selector for Nitrogen Removal and extended aeration with activated sludge recirculation **Tertiary treatment**:  Sand ﬁlter and ultraﬁltration and Disinfection with Chlorine | Al-Maadheed et al,, 2019 |
| Palestine | Nablus and Jericho Governorate | Penicillin G | 42.80 | - | Small-scale, off-grid greywater treatment systems | Craddock et al., 2020 |
| Jordan | AS-Samra WWTP | Phenazone | 70.3 | - | activated sludge/extended aeration system since 2015 | Al-Mashaqbeh et al., 2019 |
| Palestine | Nablus and Jericho Governorate | Pipemidic acid | 61 | - | Small-scale, off-grid greywater treatment systems | Craddock et al., 2020 |
| Tunisia | WWTPs Tunis-nord | Sisomycin | 42 | - | Activated sludge | Tahrani et al., 2016 |
| Tunisia | WWTPs Chotrana | Sisomycin | 57 | - | Activated sludge | Tahrani et al., 2016 |
| Tunisia | WWTPs Ben Arous | Sisomycin | 51 | - | Activated sludge | Tahrani et al., 2016 |
| Tunisia | WWTPs Charguia | Streptomycin | 56 | - | Activated sludge | Tahrani et al., 2016 |
| Tunisia | WWTPs Chotrana | Streptomycin | 50 | - | Activated sludge | Tahrani et al., 2016 |
| Jordan | AS-Samra WWTP | Sulfamethazine | 37.2 | - | activated sludge/extended aeration system since 2015 | Al-Mashaqbeh et al., 2019 |
| Saudi arabia | Two HWWTPs, Riyadh | Sulfamethoxazole |  | >98 | Hospital WWTPs  **Secondary treatment:**  Activated sludge  **Tertiary treatment**:  Sandfiltrationanddisinfection with Chlorine | Al Qarni et al., 2016 |
| Palestine | Nablus and Jericho Governorate | Sulfamethoxazole | 54.50 | - | Small-scale, off-grid greywater treatment systems | Craddock et al., 2020 |
| Tunisia | Seven Tunisian WWTPs | Sulfamethoxazole | -31 | - | Activated sludge | Moslah et al., 2017 |
| Jordan | Al-Aqaba WWTP, Jordan | Sulfamethoxazole |  | 100 | **Secondary treatment:**  Activated sludge  **Tertiary treatment**:  waste stabilization pond | Al-Tarawneh et al., 2014 |
| Jordan | Al-Salt WWTP, Jordan | Sulfamethoxazole | Concentration in Influent = 0 ng/L  Concentration in Effluent = 300 ng/L | - | Rotating biological contractors with extended aeration | Al-Tarawneh et al., 2014 |
| Jordan | AS-Samra WWTP | Sulfamethoxazole | 45.8 | - | Activated sludge/extended aeration system since 2015 | Al-Mashaqbeh et al., 2019 |
| Palestine | El Beireh | Testosterone | 100 | - | Secondary tratment: Activated sludge- Bottom aeration. | Dotan et al,, 2016 |
| Palestine | Nablus | Testosterone | 100 | - | Secondary tratment: Activated sludge- Bottom aeration. | Dotan et al,, 2016 |
| Israel | Yad Hana | Testosterone | 89.3 | 100 | Secondary treatment: Aeration pond. | Dotan et al,, 2016 |
| Israel | Ra'anana | Testosterone | 100 | - | **Secondary** treatment: Sequence bath reactor  **Tertiary** treatment: Sand filtration | Dotan et al,, 2016 |
| Israel | Ben-Gurion airport | Testosterone | 100 | - | **Tertiary treatment**: membrane bioreactor. | Dotan et al,, 2016 |
| Israel | Shafdan | Testosterone | 100 | - | **Secondary treatment**: Activated sludge/bottom aeration  **Additional treatment:** Soil-aquifer treatment | Dotan et al,, 2016 |
| Israel | HodHasharon | Testosterone | 100 | - | **Secondary treatment**:  Activated sludge/bottom aeration  **Tertiary treatment:**  Sand filtration + disinfection with Uv. | Dotan et al,, 2016 |
| Israel | Yeruham | Testosterone | 100 | - | **Secondary treatment**:  Activated sludge/Rotating disks  **Tertiary treatment**: sand filtration. | Dotan et al,, 2016 |
| Qatar | WWTP, Qatar | Tetracycline |  | 19.2 | Old WWTP  **Secondary treatment**:  Conventional aeration and activated sludge recirculation  **Tertiary treatment**:  Sand ﬁlter and Disinfection with Chlorine | Al-Maadheed et al,, 2019 |
| Qatar | WWTP, Qatar | Tetracycline |  | 0 | New WWTP  **Secondary treatment**:  Anaerobic Selector for phosphorous removal, Anoxic Selector for Nitrogen Removal and extended aeration with activated sludge recirculation **Tertiary treatment**:  Sand ﬁlter and ultraﬁltration and Disinfection with Chlorine | Al-Maadheed et al,, 2019 |
| Palestine | Nablus and Jericho Governorate | Tetracycline | -23 | - | Small-scale, off-grid greywater treatment systems | Craddock et al., 2020 |
| Jordan | AS-Samra WWTP | Thiabendazole | -17.2 | - | Activated sludge/extended aeration system since 2015 | Al-Mashaqbeh et al., 2019 |
| Tunisia | WWTPs Tunis-nord | Thiamphenicol | 100 | - | Activated sludge | Tahrani et al., 2016 |
| Palestine | Nablus and Jericho Governorate | Triclocarban | 89 | - | Small-scale, off-grid greywater treatment systems | Craddock et al., 2020 |
| Palestine | El Beireh | Triclosan | -36900 | - | Secondary tratment: Activated sludge- Bottom aeration. | Dotan et al,, 2016 |
| Israel | Yad Hana | Triclosan | 50 | 100 | Secondary treatment: Aeration pond. Water reuse: irrigation | Dotan et al,, 2016 |
| Israel | Ra'anana | Triclosan | 77 | 92 | **Secondary** treatment: Sequence bath reactor  **Tertiary** treatment: Sand filtration | Dotan et al,, 2016 |
| Israel | Ben-Gurion airport | Triclosan | 78 | 89 | Tertiary treatment: membrane bioreactor. | Dotan et al,, 2016 |
| Israel | Shafdan | Triclosan | 27 | 100 | **Secondary treatment**: Activated sludge/bottom aeration  **Additional treatment:** Soil-aquifer treatment | Dotan et al,, 2016 |
| Israel | Had Hasharon | Triclosan | 55 | 78 | Tertiary treatment + Sand filtration + disinfection with Uv. | Dotan et al,, 2016 |
| Israel | Yeruham | Triclosan | 93 | 96 | **Secondary treatment**:  Activated sludge/Rotating disks  **Tertiary treatment**: sand filtration. | Dotan et al,, 2016 |
| Jordan | AS-Samra WWTP | Trimethoprim | 97.1 | - | activated sludge/extended aeration system since 2015 | Al-Mashaqbeh et al., 2019 |
| Palestine | Nablus and Jericho Governorate | Vancomycin | 100 | - | Small-scale, off-grid greywater treatment systems | Craddock et al., 2020 |
| Palestine | Nablus and Jericho Governorate | Alachlor | -42 |  | Small-scale, off-grid greywater treatment systems | Craddock et al., 2020 |
| Palestine | Nablus and Jericho Governorate | Atrazine | 60 |  | Small-scale, off-grid greywater treatment systems | Craddock et al., 2020 |
| Palestine | Nablus and Jericho Governorate | Trifluralin | 20 |  | Small-scale, off-grid greywater treatment systems | Craddock et al., 2020 |
| Tunisia | Coastline WWTPs 1 | Benzylparaben | 95 |  | Not available | Hassine et al., 2011 |
| Tunisia | Coastline WWTPs 1 | Buthylparaben | 75 |  | Not available | Hassine et al., 2011 |
| Tunisia | Coastline WWTPs 1 | Ethylparaben | 100 |  | Not available | Hassine et al., 2011 |
| Tunisia | Coastline WWTPs 1 | Propylparaben | 67 |  | Not available | Hassine et al., 2011 |
| Tunisia | Coastline WWTPs 1 | Methylparaben | 21 |  | Not available | Hassine et al., 2011 |
| Tunisia | Coastline WWTPs 2 | Benzylparaben | 87 |  | Not available | Hassine et al., 2011 |
| Tunisia | Coastline WWTPs 2 | Buthylparaben | 100 |  | Not available | Hassine et al., 2011 |
| Tunisia | Coastline WWTPs 2 | Ethylparaben | 100 |  | Not available | Hassine et al., 2011 |
| Tunisia | Coastline WWTPs 2 | Propylparaben | 98 |  | Not available | Hassine et al., 2011 |
| Tunisia | Coastline WWTPs 2 | Methylparaben | 100 |  | Not available | Hassine et al., 2011 |
| Tunisia | Coastline WWTPs 3 | Benzylparaben | 98 |  | Not available | Hassine et al., 2011 |
| Tunisia | Coastline WWTPs 3 | Buthylparaben | 100 |  | Not available | Hassine et al., 2011 |
| Tunisia | Coastline WWTPs 3 | Ethylparaben | 100 |  | Not available | Hassine et al., 2011 |
| Tunisia | Coastline WWTPs 3 | Propylparaben | 100 |  | Not available | Hassine et al., 2011 |
| Tunisia | Coastline WWTPs 3 | Methylparaben | -240 |  | Not available | Hassine et al., 2011 |
| Tunisia | WWTP 4 (Oued Meliane) | Illicit drugs | 100 |  | Biologic-  activated  sludge | Moslah et al., 2017 |
| Tunisia | WWTP 5 (Oued Meliane) | Illicit drugs | 100 |  | Biologic-  activated  sludge | Moslah et al., 2017 |
| Tunisia | WWTP 2 (Canal El Khalij) | Illicit drugs | 100 |  | Biologic-  activated  sludge | Moslah et al., 2017 |
| Tunisia | WWTP 6 (Oued Meliane) | Illicit drugs | 100 |  | Biologic-  activated  sludge | Moslah et al., 2017 |
| Tunisia | WWTP 1 (Canal El Khalij) | Illicit drugs | 77 |  | Biologic-  activated  sludge | Moslah et al., 2017 |
| Tunisia | WWTP 2 (Canal El Khalij) | Illicit drugs | 52 |  | Biologic-  activated  sludge | Moslah et al., 2017 |
| Tunisia | WWTP 3 (Canal El Khalij) | Illicit drugs | 100 |  | Biologic-  activated  sludge | Moslah et al., 2017 |
| Tunisia | WWTP 5 (Oued Meliane) | Illicit drugs | - |  | Biologic-  activated  sludge | Moslah et al., 2017 |
| Tunisia | WWTP 6 (Oued Meliane) | Illicit drugs | 33 |  | Biologic-  activated  sludge | Moslah et al., 2017 |
| Tunisia | WWTP 1 (Canal El Khalij) | Illicit drugs | 100 |  | Biologic-  activated  sludge | Moslah et al., 2017 |
| Tunisia | WWTP 2 (Canal El Khalij) | Illicit drugs | 100 |  | Biologic-  activated  sludge | Moslah et al., 2017 |

**References** **supplementary materials**

Abbassy M.S., Ibrahim H.Z.,Abo Elamayem M. (1999). Occurrence of pesticides and polychlorinated biphenols in water of the Nile river and estuaries of Rosetta and Damietta branches, North Delta. Egypt J Environ Sci Health. 34, 255–267.

Abbassy M.S. (2000). Pesticides and polychlorinated biphenyls drained into North coast of the Mediterranean sea, Egypt. Bull Environ Contam Toxicol. 64, 508.

Abdel-Halim K.Y., Salama A.K., El-khateeb E.N., Bakry N.M. (2006). Organophosphorus pollutants (OPP) in aquatic environment at Damietta Governorate, Egypt: implications for monitoring and biomarker responses. Chemosphere 63,1491–149.

Agah H., Mehdinia A., Bastami K.D., Rahmanpour.S.(2016). Polycyclic aromatic hydrocarbon pollution in the surface water and sediments of Chabahar Bay, Oman Sea. Marine Pollution Bulletin. (In press.)

Aliabad M.K., Nassiri M., Kor K. (2019). Microplastics in the surface seawaters of Chabahar Bay, Gulf of Oman (Makran Coasts). Mar. Pollut. Bull. 143, 125–133. https://doi.org/10.1016/j.marpolbul.2019.04.037.

Alquwaizany A.S., Alfadul S.M., Khan M.A., Alabdulaaly A.I. (2019). Occurrence of organic compounds in groundwater of Saudi Arabia. Environ Monit Assess.191, 601.

Al-Rubaie K.H., Fenjan A.M., Mahmood W.A. (2012). Evaluation of the Organic-Pollution Based on the Determination of some Polycyclic Aromatic Hydrocarbons (PAHs) in Tigris River Water in 2012 at Baghdad City , Iraq. Baghdad Science Journal. 11(2).

Amine H., Gomez E., Halwani J., Casellas C., Fenet H. (2012). UV filters, ethylhexyl methoxycinnamate, octocrylene and ethylhexyl dimethyl PABA from untreated wastewater in sediment from eastern Mediterranean river transition and coastal zones. Marine Pollution Bulletin 64, 2435–2442.

Aranami K., Readman J W. (2007). Photolytic degradation of triclosan in freshwater and seawater. Chemosphere 66, 1052–1056.

Barakat A.O. (2004). Assessment of persistent toxic substances in the environment of Egypt. Environ Int. 30, 309–322.

Barhoumi B., Le Menach K., Dévier M.H., El megdiche Y., Hammami B., Ben Ameur W., Ben Hassine S., Cachot J., Budzinski H., Driss M.R.(2013). Distribution and ecological risk of polychlorinated biphenyls (PCBs) and organochlorine pesticides (OCPs) in surface sediments from the Bizerte lagoon, Tunisia. Environmental science and pollution research online, ISSN 1614–7499.

Basim .Y. Al-Khafaji , Afrah. A. Maktoof and Rasha. S. Nuhair. (2014). Concentration of organochlorine pesticide residues in water, sediment and fish from the Euphrates River near the center of Al-Nassiriyia city, Iraq. MARSH BULLETIN 9(2) 2014.

[_Bazin I_](https://www.ncbi.nlm.nih.gov/pubmed/?term=Bazin%20I%5BAuthor%5D&cauthor=true&cauthor_uid=22947508).,[_IbnHadjHassine A_](https://www.ncbi.nlm.nih.gov/pubmed/?term=Ibn%20Hadj%20Hassine%20A%5BAuthor%5D&cauthor=true&cauthor_uid=22947508).,[_Haj Hamouda Y_](https://www.ncbi.nlm.nih.gov/pubmed/?term=Haj%20Hamouda%20Y%5BAuthor%5D&cauthor=true&cauthor_uid=22947508).,[_Mnif W_](https://www.ncbi.nlm.nih.gov/pubmed/?term=Mnif%20W%5BAuthor%5D&cauthor=true&cauthor_uid=22947508).,[_Bartegi A_](https://www.ncbi.nlm.nih.gov/pubmed/?term=Bartegi%20A%5BAuthor%5D&cauthor=true&cauthor_uid=22947508),.[_Lopez-Ferber M_](https://www.ncbi.nlm.nih.gov/pubmed/?term=Lopez-Ferber%20M%5BAuthor%5D&cauthor=true&cauthor_uid=22947508).,[_De Waard M_](https://www.ncbi.nlm.nih.gov/pubmed/?term=De%20Waard%20M%5BAuthor%5D&cauthor=true&cauthor_uid=22947508).,[_Gonzalez C_](https://www.ncbi.nlm.nih.gov/pubmed/?term=Gonzalez%20C%5BAuthor%5D&cauthor=true&cauthor_uid=22947508).(2012).Estrogenic and anti-estrogenic activity of 23 commercial textile dyes. Ecotoxicol Environ Saf. 85, 131-136.

Belhaj D., Jaabiri I., Ayadi H., Kallel M., Zhou J.L. (2014a).Occurrence and removal of steroidal estrogens in Centre Eastern Tunisia municipal sewage treatment plant. Desalin Water Treat 52, 2330–2339.

Belhaj D., Turki N., Jaabiri I.,Kallel M., Ayadi H., Zhou J.L.(2014b).Comparison of estrogen compounds removal efﬁciency in sample and alternating anoxic/aerobic activated sludge process. J Environ Sci Toxicol Food Technol. 8, 100–108. Belver C., .Bedia J., Rodriguez J.J.(2016). Zr-doped TiO2 supported on delaminated clay materials for solar photocatalytic treatment of emerging pollutants.Journal of Hazardous Materials. 322,233-242.

Ben Fredj F., Irie M., Han J. Limam A., Gharbi A., Isoda H. (2012). Sensitivity of in vitro bioassays towards several water origins in Tunisian arid and semi-arid area. J Arid Land Stud. 22,–1, 319–322.

Berna J.L., Ferrer J., Moreno A., Prats D., Bevia F.R. (1989). The fate of LAS in the environment. Tenside Surfactants Deterg.26:101–7.

Bettiche F., Grunberger, O., Belhamra, M., 2017. Water contamination by pesticides under intensive production system (greenhouses), case of Biskra, Algeria. Courrier du Savoir, 23, 39-48.

Bidlack H. D., (1979). Degradation of Chlorpyrifos in Soil Under Aerobic, Aerobic/Anaerobic, and Anaerobic Conditions. Dow Chemical U.S.A., unpublished report GHC-1258.

Bisson M., Doornaert B., Hulot C., Joachim S., Lacroix G., Lefevre J P., Malleret L., Tissot S. (2005). benzo(b)fluoranthène INERIS - Fiche de données toxicologiques et environnementales des substances chimiques. 36p.

Cardinal P., Anderson J C., Carlson J C, Low J E., Challis J K., Wong C S., Hanson M L. (2016). Late season pharmaceutical fate in wetland mesocosms with and without phosphorous addition. Environmental Science and Pollution Research. 23, 22678–22690.

Carr D. L., Morse A. N., Zak J. C., Anderson T. A. (2010). Microbially mediated degradation of common pharmaceuticals and personal care products in soil under aerobic and reduced oxygen conditions. Water, Air, & Soil Pollution, 216, 633–642.

Cary L., Casanova J., Gaaloul N., Guerrot C. (2013) Combining boron isotopes and carbamazepine to trace sewage in salinized groundwater: A case study in Cap Bon, Tunisia. Applied Geochemistry 34, 126–139.

Castro T.F., Yoshida T. (1971). Degradation of organochlorine insecticides in flooded soils in the Philippines. J. Agric. Food Chem. 19, 1168–1170.

Chaib, O., Arhoune, B., Achour, S., Moreau-Guigon, E., Alliot, F., Chevreuil, M., El Fakir, S., El Arabi, I., Oumokhtar, B., 2019. Occurrence and seasonal variation of antibiotics in FezMorocco surface water. Am. J. Environ. Sci. 15, 127–136. https://doi.org/10.3844/ ajessp.2019.127.136.

Chbib C., NET S., Hamzeh M., Dumoulin D., Ouddane B., Baroudi M., (2017). Assessment of pesticide contamination in Akkar groundwater, northern Lebanon. Environ. Sci. Pollut. Res. 25.

Chen Z Y., Liu C., Lu Y H., Yang L L., Li M., He M D., Chen C H., Zhang L., Yu Z P., Zhou Z. (2016). Cadmium Exposure Enhances Bisphenol A-Induced Genotoxicity through 8-Oxoguanine-DNA Glycosylase-1 OGG1 Inhibition in NIH3T3 Fibroblast Cells. Cellular physiology and biochemistry : international journal of experimental cellular physiology, biochemistry, and pharmacology 39, 3, 961–974.

Chen W., Xu J., Lu S., Jiao W., Wu L., Chang A. C. (2013). Fates and transport of PPCPs in soil receiving reclaimed water irrigation. Chemosphere, 93, 2621–2630.

Council of the European Economic Community Directive (1980) Council of the European Economic Community Directive 80/778/EEC (1980).. On the quality of water intended for human consumption. (OJ No.229, 30.08.1980, p. 30).

Council of the European Economic Community Directive (1998) Council of the European Economic Community Directive 98/83/EEC (1998). On the quality of water intended for human consumption. The Council of the European Communities. (OJ No. L330, 5.12.1998, p. 32).

Coover M.P., Sims R.C.C. (1987). The effects of temperature on polycyclic aromatic hydrocarbon persistence in an unacclimated agricultural soil. Haz. Waste Haz. Mat. 4, 69–82.

Dutch DWD (Dutch Drinking Water Directive) (2011) (Besluit van 23 mei 2011, houdende bepalingen inzake de productie en distributie van drinkwater en de organisatie van de openbare drinkwatervoorziening; Drinkwaterbesluit). Staatsblad van het Koninkrijk der Nederlanden, 293 (in Dutch). http://www.who.int/water_sanitation_health/publications/2011/dwq_chapters/en/index.html

Durán-Álvarez J. C., Prado-Pano B., Jiménez-Cisneros B. (2012). Sorption and desorption of carbamazepine, naproxen and triclosan in a soil irrigated with raw wastewater: Estimation of the sorption parameters by considering the initial mass of the compounds in the soil. Chemosphere, 88, 84–90.

ECCO. (1997). Monograph on the Review of Aldicarb European Commission Peer Review Programme.

El Bakouri H., Ouassini A., Morillo J., Usero J. (2008). Pesticides in ground water beneath Loukkos perimeter, Northwest Morocco. Journal of Hydrology. 348, 270-278.

El Barbary A.A., El Bouraie M.M.,Yehia M.M. (2008). Evaluation of organochlorine pesticides (OCPS) in surface water and bed sediment samples from the River Nile at Rosetta Branch, Egypt. J. Appl. Sci. Res. 4, 1985–1993.

El-Gendy K.S., Abd-Allah A.M., Ali H.A., Tantawy G., El-Sebae A.E. (1991) Residue levels of chlorinated hydrocarbons in water and sediment samples from Nile Branches in the Delta, Egypt. J Environ Sci Health. 26,15–36.

El-Kabbany S., Rahed M.M., Zayed M.A. (2000). Monitoring of pesticide levels in some water supplies and agricultural land in El-Haram, Giza. J Hazard Mater. 72, 11–21.

El-Osmani R., NetT S., Dumoulin D., Baroudi M., BakkourH., Ouddane B. (2014).Solid phase extraction of organochlorine pesticides residues in groundwater (Akkar plain, north Lebanon). Int. J. Environ. Res. 8 (4), 903-912.

El-Saeid M.H., Al-TurkiA.M,.Al-Wable M.I., Abdel-Nasser G. (2011). Evaluation of pesticide residues in Saudi Arabia ground water. Research Journal of Environmental Sciences5 (2): 171–178.

EI-Sebae A.H., Abo-Elamayem M. (1978). A survey of expected pollutants drained to the Mediterranean in the Egyptian Region. Proc. of the XXXVI Congress and Plenary Assembly of the Internat. Comm. of Sci. Explor. of the Mediterranean Sea, Antalya, Turkey, pp. 149–153.

Ettinger M B., Ruchhoft C C. (1950). Persistence of monochlorophenols in polluted river water and sewage dilutions. U.S. Pub. Health Serv., Environ. Health Center, Cincinnati, Ohio.

Ettore C. (2008). Pesticide Risk Assessment in Rice Paddies Theory and Pratice. Pesticide Risk Assessment in Rice Paddies. Chapter 4 - Water Resource Contamination in Italian Paddy Areas. 59-67.

European Chemicals Bureau. (2007). European Union Summary Risk Assessment Report. Benzene. Final Approved Version.

European Union 80/778/EEC. Council directive of 15 July 1980 relating to the quality of water intended for human consumption. Official Journal of the European Communities 1980: L229/11- L229/29.

European Union 98/83/EEC. Official Journal of the European Communities 5 December 1998. Council Directive 98/83/EC of 3 November 1998 on the quality of water intended for human consumption. L330/32-330/54.

Fenet H., Mathieu O., Mahjoub O., Li Z., HillaireBuys D., Casellas C., Gomez E. (2012). Carbamazepine, carbamazepine epoxide and dihydroxycarbamazepine sorption to soil and occurrence in a wastewater reuse site in Tunisia. Chemosphere 88: 49–54.

Fono L J., Kolodziej E P., Sedlak D L. (2006). Attenuation of wastewater-derived contaminants in an effluent-dominated river. Environ. Sci. Technol., 40, 7257-7262.

Franse and de Voogt, (1997). Oestrogene verbindingen in het Nederlands milieu, MTC report.

Gan D.R., Berthouex P.M. (1994). Disappearance and crop uptake of PCBs from sludge-amended farmland. Water Environ. Res. 66: 54–69.

Gdara I., Zrafi I., Balducci C., Cecinato A., Ghrabi A. (2020). First investigation of seasonal concentration behaviors and sources assessment of aliphatic hydrocarbon in waters and sediments from Wadi El Bey, Tunisia. Archives of Environmental Contamination and Toxicology <https://doi.org/10.1007/s00244-019-00669-y>.

Greve. (1996). (Dutch Health Council). Hormoon-verstorende stoffen in Nederland. Gebruik, emissie, milieuconcentraties en fysisch/chemische karakteristieken.

Hassan F.M,, Al-Obaidy A..M.J., Salman J.M., Abdulameer S.H. (2019). Distribution of Polycyclic Aromatic Hydrocarbons in Water and Sediments in the Euphrates River, Iraq. Iraqi journal of science. Iraqi Journal of Science. 60, 12, 2572-2582.

_Hazardous Substances Data Bank (HSDB)_. <https://pubchem.ncbi.nlm.nih.gov/source/hsdb/6292> _(accessed August 2021)_.

Howard P.H. (1991). Handbook of environmental fate and exposure data for organic chemicals: Pesticides. CRC press.

Howard P H., Boethling R S., Jarvis W F., Meylan W M., Michalenko E M. (1991). Handbook of environmental degradation rates. Chealsea, Michigan, Lewis Publisher, p 725, Printup HT Ed.

IARC. (1991). Occupational Exposures in Insecticide Application, and Some Pesticides. 53, 371-402.

_INERIS. (2005). Heptachlore_.

Jalal A., Al-Tabbal,Kamel K., Al-Zboon.(2012). Suitability assessment of groundwater for irrigation and drinking purpose in the northern region of Jordan. Volume: 5 : 5, 274–290.

Jazza S.H., Abdul-Hussain Y., Al-Adhub A.Y., Al-Saad H.T. (2016a) Polycyclic Aromatic Hydrocarbons (PAHs) in water of Al-Kahlaa River in Missan Province, Iraq. ILMU KELAUTAN March, 1-8.

Kearney P.C., Nash R.G., Isensee A.R. (1969). Persistence of pesticides in soil. Chemical Fallout: Current Research on Persistent Pesticides. Miller, M.W. and Derg, C.C., Eds. Springfield, IL: Charles C. Thomas. Chpt.3.54–67.

Keck J., Sims R.C., Coover M., Park K., Symons B. (1989). Evidence for cooxidation of polynuclear aromatic hydrocarbons in soil. Wat. Res. 21,1467–1476.

Khalaf S.M., Hassan F.M., Al-Obaidy A.H.J. (2019). Detection of polycyclic aromatic hydrocarbons compounds concentrations and their fate in tigris river within Baghdad city – Iraq. Iraqi Journal of Agricultural Sciences. 1029:50(Special Issue):231- 244.

Khaled‑Khodja S., Karima Rouibah K. (2018). Selected organic pollutants (PAHs, PCBs) in water and sediments of Annaba Bay, Algeria. Euro-Mediterranean Journal for Environmental Integration (2018) 3:23.

Kolbe A., Bernasch A., Stock M., Schütte H. R., Dedek W. (1991). Persistence of the insecticide Dimethoate in three different soils under laboratory conditions. Bull. Environ. Contam. Toxicol. 46, 492–498.

Kouzayha A., Al Ashi A., Al Akoum R., Al Iskandarani M., Budzinski H., Jaber F. (2013). Occurrence of pesticide residues in Lebanon's water resources. Bull. Environ. Contam. Toxicol. 91 (5), 503-509.

Kazour, M., Jemaa, S., Issa, C., Khalaf, G., Amara, R., 2019. Microplastics pollution along the Lebanese coast (Eastern Mediterranean Basin): occurrence in surface water, sediments and biota samples. Sci. Total Environ. 696, 133933. https://doi.org/10.1016/j.scitotenv.2019.133933.

Al-Tarawneh I, El-Dosoky M, Alawi M, Batarseh M, Widyasari A, Kreuzig R, Bahadir, M (2014) Studies on human pharmaceuticals in Jordanian wastewater samples. CLEAN ± Soil, Air, Water 43: 504–511.

Lamm A., Gozlan I., Rotstein A., Avisar D.(2009). Detection of moxicillindiketopiperazine-20, 50 in wastewater samples. J Environ Sci and Health Part A 44 (14), 1512–1517. Laskowski D. A., Swann R. L., McCall P. J., Bidlack H. D. (1983). Soil Degradation Studies. Residue Rev. 85, 139–147.

Lester Y., Mamane H., Zucker I., Avisar D. (2013). Treating wastewater from a pharmaceutical formulation facility by biological process and ozone. Water Res. 47 (13), 4349–4356.

Li J.Y., Dodgen L., Ye Q.F., Gan J. (2013). Degradation kinetics and metabolites of carbamazepine in soil. Environ. Sci. Technol. 47, 3678–3684.

Lichtenstein E.P., Schulz K.R. (1959). Persistence of some chlorinated hydrocarbon insecticides influenced by soil types, rates of application and temperature. J. Econ. Entomol. 52, 124–131.

Liu F., Ying G. G., Yang J. F., Zhou L. J., Tao R., Wang L., Zhang L. J., Peng P. A. (2010). Dissipation of sulfamethoxazole, trimethoprim and tylosin in a soil under aerobic and anoxic conditions. Environmental Chemistry, 7, 370–376.

Lyman W J, Reehl W J, Rosenblatt D H. (1982). Handbook of chemical property estimation methods: environmental behaviour of organic compounds. McGraw-Hill, New York.

Mackay D., Shiu WY., Ma K C. (1992). Illustrated handbook of physical-chemical properties and environmental fate for organic chemicals. CRC. Press. Boca Raton, FL., vol. II.

Mahjoub O., Leclercq M., Bachelot M.Casellas C., Escande A., Balaguer P., Bahri A.,Gomez E.,Fenet H.(2009).Estrogen, aryl hysdrocarbon and pregnane X receptors activities in reclaimed water and irrigated soils in Oued Souhil area (NabeulTunisia). Desal 246, 425–434.

Mahjoub O., Escande A., Rosain D., Casellas C., Gomez E., Fenet H.(2011).Estrogen-like and dioxin-like organic contaminants in reclaimed wastewater: transfer to irrigated soil and groundwater. Water SciTechnol 63,1657–1662.

Malhat F., Nasr I. (2013). Monitoring of organophosphorous pesticides residues in water from the Nile River tributaries, Egypt. Am J Water Resour. 1,1–4.

Meikle R. W., Hedlund R. T. (1973). Preliminary Estimate of the Degradation Rate of Chlorpyrifos in Soil Obtained by Computer Simulation of a Simplified Kinetic Model. Dow Chemical U.S.A., unpublished report GS-1305.

Moursy A., Ibrahim M.B. (1999). Monitoring of organochlorine pollutants in the water of Lake Manzala. WEFTEC’99; 72nd Annual Conference Exposition, Cairo, Egypt; 1999.

Mzoughi N., Chouba L. (2011). Distribution and partitioning of aliphatic hydrocarbons and polycyclic aromatic hydrocarbons between water, suspended particulate matter, and sediment in harbours of the West coastal of the Gulf of Tunis (Tunisia). Journal of Environmental Monitoring 13, 689–698.

Nash R.G., Woolson E.A., (1967). Persistence of chlorinated hydrocarbon insecticides in soils. Science. 157: 924–927.

Necibi M., Saadaoui H., Atayat A., Mzoughi N. (2020). Determination of Triazole Pesticides in the Surface Water of the Medjerda River, Tunisia. Analytical Letters. DOI: 10.1080/00032719.2020.1780250.

Picó, Y., Alvarez-Ruiz, R., Alfarhan, A.H., El-Sheikh, M.A., Alshahrani, H.O., Barceló, D., 2020. Pharmaceuticals, pesticides, personal care products and microplastics contamination assessment of Al-Hassa irrigation network (Saudi Arabia) and its shallow lakes. Sci. Total Envirn. 701, 135021. <https://doi.org/10.1016/j.scitotenv.2019.135021>.

RIWA. (1998). Xeno-oestrogenen en drinkwater(bronnen).

Rodríguez-Gil J.L., Cáceres N., Dafouz R., Valcarcel Y. (2018). Caﬀeine and paraxanthine contamination in aquatic systems: Global exposure distributions and probabilistic risk assessment. Sci. Total Environ. 612, 1058–1071.

Rousseau A N., Grenier M., Lafrance P., Quilbé R. (2007). Classification des pesticides à l’aide de cartes auto-organisatrices de Kohonen en vue du développement de normes de performance agro-environnementale atteignables (NPA) à l’échelle des bassins versants. Rapport N° R-932. INRS-ETE, Québec, Canada.

Royal Society of Chemistry. (1991). The agrochemicals handbook, 3rd ed. Cambridge.

Salem A.A., Wasfi I.A., Al-Nassibi S.S. (2012). Trace determination of β-blockers and β2-agonists in distilled and waste-waters using liquid chromatography–tandem mass spectrometry and solid-phase extraction. Journal of Chromatography B, 908(0), 27–38.

Samia K., Dhouha A., Anis C., Ammar M., Rim A., Abdelkrim C.(2018). Assessment of organic pollutants (PAH and PCB) in surface water: sediments and shallow groundwater of Grombalia watershed in northeast of Tunisia. Arabian Journal of Geosciences 11, 34.

Saouli C., Assabaa R. (2018). Behavior of pharmaceuticals in effluent water: Saidal Group (Constantine Algeria). Rasayan J. Chem. 11, 951–954. https://doi.org/10.31788/ RJC.2018.1133008.

Scully Jr F E., Hoigne J. (1987) Rate Constants for reactions of singlet oxygen with phenols and. other compounds in water. Chemosphere. 16, 681-694.

Sedlak D L., and Andren A W. (1991). Aqueous-phase oxidation of polychlorinated biphenyls by hydroxyl radicals. Environ. Sci. Technol. 25:1419-1427.

Smith A E., Aubin A J. (1991). Metabolites of [C-14] 2,4-dichlorophenoxyacetic acid in Saskatchewan soils. J. Agric. Food Chem. 39, 11, 2019– 2021, DOI: 10.1021/jf00011a029.

Symons B.D., Sims R.C. Grenney W.J. (1988). Fate and transport of organics in soil: model predictions and experimental results. JWPCF. 60, 1684–1693.

Tabak H H., Quave S A., Mashni C I., Barth E F. (1981). Biodegradability studies with organic priority pollutant compounds. J Water Pollut Contr Fed, 53, 10, 1503-1518.

Tahrani L., Mehri I., Reyns T., Anthonissen R., Verschaeve L., Bel Haj Khalifa A., Van Loco J., Abdenaceur H., Ben Mansour H. (2017). UPLC-MS/MS analysis of antibiotics in pharmaceutical effluent in Tunisia: ecotoxicological impact and multi-resistant bacteria dissemination. Archives of Microbiology. doi.org/10.1007/s00203-017-1467-x.

Tasfaout A., Youcef H., Naas K., Chefirat B., Rezkallah H. (2018). Evaluation of Organochlorine Pesticide Residues in Undergroundwater of the Mostaganem Region, Algeria. Journal of Medical Toxicology Research. J Med Toxicol Res, 1:1.

Tixier C., Singer H P., Oellers S., Müller S R. (2003). Occurrence and fate of carbamazepine, clofibric acid, diclofenac, ibuprofen, ketoprofen, and naproxen in surface waters. Environ. Sci. Technol., 37, 1061-1068.

Toumi.H.,Abidli S., Bejaoui M. (2019). Microplastics in freshwater environment: the first evaluation in sediments from seven water streams surrounding the lagoon of Bizerte (Northern Tunisia). Environmental Science and Pollution. Research. 26, 14673-14682.

ToxNet. (2016). 2,4,6-Trichlorophenol. Toxicology data network. Bethesda (MD), USA: United States National Library of Medicine. Available from: https://toxnet. nlm.nih.gov, accessed August 2021.

U.S. Environmental Protection Agency. (1988). Method 525. Determination of organic compounds in drinking water by liquid-solid extraction and capillary column gas chromatography/mass spectrometry. In: Methods for the determination of organic compounds in drinking water. Cincinnati, OH, Environmental Monitoring Systems Laboratory, 1988:325-356 (EPA Report No. EPA-600/4-88/039; US NTIS PB89-220461).

U.S. National Library of medicine, Hazardous Substances Databank, 1995; Oregon State University, extension Toxicology network (base de données), 1995 et Bristish Crop Protection Council, The Pesticide Manual, différentes éditions.

Vozhzennikov О I., Bulgakov A A., Popov V E., Lukoyanov N F., Naidenov A V., Burkov A.J., Kutnyakov J V., Zhirnov V G. (1997). Review of migration and transformation parameters of selected POPs, EMEP/ Meteorological Synthesizing Centre - East Report 3/97.

Wakkaf T., El Zrelli R., Kedzierski M., Balti R., Shaiek M., Mansour L., Tlig-Zouari S., Bruzaud S., Rabaoui L. (2020). Characterization of microplastics in the surface waters of an urban lagoon (Bizerte lagoon, Southern Mediterranean Sea): Composition, density, distribution, and influence of environmental factors. Marine Pollution Bulletin, 160, 111625.

Waters J, Holt M.S, Matthijs E. (1989). Fate of LAS in sludge amended soils. Tenside Surfactants Deterg .26:129–35.

Wauchope R.D., Buttler T.M., Hornsby A.G. Augustijn Beckers P.W.M., Burt, J.P. (1992). SCS/ARS/CES Pesticide properties database for environmental decision making. Rev. Environ. Contam. Toxicol., 123, 6–15.

Wild S.R., Obbard J.P., Munn C.I., Berrow M.L. Jones K.C. (1991). The long-term persistence of polynuclear aromatic hydrocarbons (PAHs) in an agricultural soil amended with metal-contaminated sewage sludges. Sci. Total Environ. 10, 235–253.

WHO (World Health Organization) (2003) Background document for preparation of WHO Guidelines for drinking-water quality. Geneva, World Health Organization.

WHO (World Health Organization) (2004) Background document for preparation of WHO Guidelines for drinking-water quality. Geneva, World Health Organization.WHO (World Health Organization). 2011. Guidelines for Drinking Water Quality, fourth ed. WHO, Geneva, World Health Organization.

World Health Organization Regional Office for the Eastern Mediterranean Regional Centre for Environmental Health Activities CEHA (2006) A compendium of drinking-water quality standards in the Eastern Mediterranean Region.

Xu J., Wu L., Chang A. C. (2009). Degradation and adsorption of selected pharmaceuticals and personal care products (PPCPs) in agricultural soils. Chemosphere, 77, 1299–1305.

Yamashita N., Urushigawa Y., Masunaga S., Walash M., Miyazaki A. (2000). Organochlorine pesticides in water; sediment and fish from the Nile River and Manzala Lake in Egypt. Int. J. Environ. Anal. Chem. 77, 289–303.

Yamamoto H., Nakamura Y., Moriguchi S., Nakamura Y., Honda Y., Tamura I., Hirata Y., Hayashi A., Sekizawa J. (2009). Persistence and partitioning of eight selected pharmaceuticals in the aquatic environment: Laboratory photolysis, biodegradation, and sorption experiments. Water Res., 4, 351-362.

Younes H.A., Mahmoud H.M., Abdelrahman M.M., Nassar, H.F. (2019). Seasonal occurrence, removal efficiency and associated ecological risk assessment of three antibiotics in a municipal wastewater treatment plant in Egypt. Environ. Nanotechnology, Monit. Manag. 12 100239. <https://doi.org/10.1016/j.enmm.2019.100239>

Ying G.G., YU X.Y., Kookana R.S. (2007). Biological degradation of triclocarban and triclosan in a soil under aerobic and anaerobic conditions and comparison with environmental fate modeling Environmental Pollution. 150, 300–305.

Zayen A., Sayadi S., Chevalier C., Boukthir M., Ismail S.B., Tedetti, M. (2020). Microplastics in surface waters of the Gulf of Gabes, southern Mediterranean Sea: distribution, composition and influence of hydrodynamics. Estuar. Coast. Shelf Sci. 242, 106832. https://doi.org/10.1016/j.ecss.2020.106832

Zoller U., Romano R. (1984). Nonionic surfactants in municipal sewage in Israel. J. Am. Oil Chem. Soc. 65(5), 971–976.

Zoller U. (1985). The "hard" and "soft" surfactant profile of Israel municipal wastewaters. J. Am. Oil Chem. Soc. 62(6), 1006–1009.

Zoller U. (1989). Nonionic surfactants in household detergents and their distribution in the environment. Tenside Surfact. Deter. 26(6), 394–399.

Zoller U. (1992). Distribution and survival of nonionic surfactants in the surface sea and groundwater of Israel. J. envir. Sci. Hlth A27(6), 1521–1533.

Zoller U., Romano R. (1983). Determination of nonionic detergents in municipal wastewater. Environ. Int. 9, 55451.

Zoller U., Ashash E., Ayali G., Shafir S. (1990). Nonionic detergents as tracers of groundwater pollution caused by municipal sewage. Envir. lnm. 16, 301–306.

Zoller U. (2006). Water reuse/recycling and reclamation in semiarid zones: The Israeli case of salination and “hard” surfactants pollution of aquifers. J. Environ. Eng. 132, 683–688.

Zoller U.,Ashash E., Ayali G., Shafir S., Azmon B. (1990). Nonionic surfactants as tracers of ground water pollution caused by municipal sewage. Environ. Int. 163, 301–306.

Zoller, U. (1994). Groundwater contamination by surfactants. Pages 273-292 in Groundwater contamination and control (U. Zoller, ed.,) Marcel Dekker: New York.

Zou H, Radke M, Kierkegaard A, MacLeod M, McLachlan M S. (2015). Environ. Sci. Technol., 49, 1646-1653.
